# Power quality enhancement in wind energy applications through E^2^PLL-based DSTATCOM control

**DOI:** 10.1371/journal.pone.0355943

**Published:** 2026-08-21

**Authors:** Ali Sait Özer, Fehmi Sevilmiş, Hulusi Karaca, Hafiz Ahmed

**Affiliations:** 1 Department of Control and Automation Technology, Konya Technical University, Konya, Türkiye; 2 Department of Electrical and Electronics Engineering, Selçuk University, Konya, Türkiye; 3 School of Electrical and Electronic Engineering, The University of Sheffield, Sheffield, United Kingdom; 4 Autonex Systems Limited, Coventry, United Kingdom; Wuhan University, CHINA

## Abstract

The distribution static compensator (DSTATCOM) is widely employed to regulate the voltage of self-excited induction generators (SEIGs) in wind energy systems. It supplies the reactive power demanded by both the SEIG and the connected load, drawing on the DC bus voltage for this purpose. Effective operation requires maintaining the DC bus voltage at a constant reference value and accurately determining the reactive power demand. This, in turn, depends on precise estimation of the reference source currents. Under nonlinear and unbalanced loading conditions, inadequate filtering of load currents can lead to errors in separating their active and reactive components, thereby degrading system performance. To address this challenge, this paper proposes an extended enhanced phase-locked loop (E^2^PLL)-based control algorithm for DSTATCOM-supported SEIGs operating with nonlinear and unbalanced loads. The proposed E²PLL achieves an effective balance between structural complexity and filtering capability, enabling accurate estimation of load current magnitude and frequency. This allows the DSTATCOM to maintain balanced and sinusoidal source currents even under nonlinear and unbalanced loading conditions. The proposed approach is comprehensively validated through real-time hardware-in-the-loop system (OPAL-RT) under these adverse conditions. In addition, robustness evaluations under practical operating disturbances, including wind-speed variations, measurement noise, and DC-link voltage ripple, further demonstrate the reliability of the proposed controller. The results demonstrate its effectiveness in ensuring accurate current estimation and stable system operation.

## Introduction

Wind energy has become one of the most affordable and reliable renewable energy options across the world. This has led to a significant research effort in recent years in wind energy integrated energy systems [1–3]. Large turbines are already helping significantly cut carbon emissions from the main power grid. According to Wind Europe, Europe has seen 16.4 GW of wind turbine installation in 2024, and it is expected that the total installation will be roughly 450 GW by 2030. Despite this progress in large wind turbines, these turbines are not suitable in all scenarios. Small turbines could reshape energy use in remote or off-grid communities where large turbines are not feasible. For these smaller systems, self-excited induction generators (SEIGs) are often seen as a good fit. They’re simple to build, sturdy, and cheaper to maintain. However, these advantages come with the challenge of controlling the system itself in diverse operating conditions. SEIGs struggle to keep voltage and frequency stable under changing conditions, and the problem gets worse when loads introduce harmonics, which drag down performance even further [4].

The voltage regulation challenges of SEIGs originate from their dependence on reactive power to sustain self-excitation. In islanded operation, particularly in remote or off-grid locations, this reactive power is supplied through a variable capacitor bank. While this arrangement enables autonomous operation, it also makes the generator highly sensitive to variations in load conditions. The presence of load-induced harmonics further aggravates the situation, leading to poor voltage and frequency stability and, ultimately, degraded power quality in stand-alone SEIG-based wind energy systems. Passive filtering, typically implemented using networks of resistors, inductors, and capacitors, has been employed to alleviate these issues. Such filters are relatively simple and cost-effective, but they introduce several technical drawbacks. They increase the overall size and weight of the system, reduce efficiency due to inherent losses, and carry the risk of resonance under certain operating conditions. These limitations restrict their suitability for small-scale and remote applications, where compactness, reliability, and efficiency are critical. Consequently, researchers have shifted attention toward active filtering solutions, with the distribution static compensator (DSTATCOM) emerging as a promising alternative [5,6].

In SEIG-based energy conversion systems, the primary sources of anomalies are fluctuations in terminal voltage and distortions in load current harmonics. A DSTATCOM with custom controller addresses these issues by monitoring these two signals and generating the necessary compensation to provide reactive power support for the SEIG while maintaining balanced source currents under distorted load conditions. To detect these anomalies, the phase-locked loop (PLL) is widely employed in the literature. For example, in [7], a conventional synchronous reference frame PLL (SRF-PLL) is combined with a low-pass filter (LPF) to regulate the DSTATCOM. However, the SRF-PLL demonstrates limited resilience to harmonics owing to the dynamic response vs. disturbance rejection trade-off in PLL tuning, and the accompanying LPF is also constrained by its non-ideal behavior in practice. A similar LPF-based approach is reported in [8], which also suffers from the same issue. In [9], an alternative method is presented that utilizes a Kalman filter for SEIG terminal voltage estimation, coupled with a cascaded proportional-integral (PI) and proportional-resonant (PR) control strategy for DSTATCOM operation. While this technique enhances monitoring and control accuracy, it requires extensive parameter tuning, which becomes difficult to manage across a wide range of operating scenarios.

The work in [10] improves upon the limitations of a conventional PI controller by integrating an adaptive neuro-fuzzy inference system (ANFIS). In this scheme, the time derivative of the tracking error is used as an ANFIS input; however, this can degrade system performance under noisy measurement conditions, as differentiation tends to amplify noise. In the literature, a filtering differentiator is often recommended to address this issue, which comes with additional tuning complexity. A generalized integrator observer (GIO) is proposed in [11] to achieve effective band-pass filtering, but its performance deteriorates in the presence of sub- and low-order harmonics or measurement offsets [12]. In low-cost systems such as small-scale SEIG, this imposes additional constraints by requiring expensive instrumentation and data processing systems. This issue is addressed in [13] by proposing a dual third-order generalized integrator (TOGI) approach, which completely eliminates the DC offset in the load current measurement. Despite TOGI’s excellent performance, it cannot fully remove odd-order harmonics; a moving-average filter (MAF) can address this. Additionally, [13] uses a PI-type loop filter, which can slow the PLL’s dynamic response; in contrast, a quasi type‑1 PLL uses only a proportional loop filter while offering type‑2 performance without integral gain. In [14], a modified generalized integrator combined with a frequency-locked loop (FLL) is proposed; it rejects DC offset while achieving good dynamic performance thanks to the FLL. Like [13], this approach cannot fully eliminate odd-order harmonics at the nominal frequency (unlike a moving-average filter), so there is room to improve harmonic robustness and thereby the overall efficiency of the SEIG system.

Similarly, [15] introduces an enhanced PLL (EPLL) for estimating the total peak value of the phase voltage, which is then used to generate DSTATCOM switching pulses. The drawback of this approach lies in the requirement of implementing EPLL separately for each phase, thereby increasing real-time computational complexity, which may necessitate the use of high-performance and expensive embedded system for the control purpose.

To overcome the limitations of conventional integer-order PI controllers, a fractional-order PI controller for SEIG-based isolated power systems was proposed in [16]. To further enhance controller performance, particle swarm optimization (PSO) was employed for parameter tuning. This approach relies on system identification for tuning, which uses the current system parameters. However, as the system ages and parameters change, the optimized settings gradually become suboptimal, leading to performance degradation. A related method using dragonfly optimization was presented in [17], which suffers from the same issue. For a broader comparison of evolutionary algorithm-based optimization techniques for SEIG controller parameter tuning, reference [18] provides a detailed assessment.

In [19], the authors addressed a separate but important challenge in SEIG control: ensuring secure operation when communication links between sensors and the control system are compromised. They proposed a chaos-based encryption strategy to safeguard control signals, thereby enabling secure SEIG operation.

To mitigate load harmonics, [20] introduced a delayed signal generation technique combined with a low-pass filter (LPF) to achieve harmonic-resilient signal extraction in STATCOM-based SEIG control. While this fixed-delay method performs well under nominal conditions, deviations from nominal frequency introduce steady-state errors that reduce system performance, thereby making the system operation sub-optimal. In recent years, disturbance observers (DOs) have gained attention for renewable energy systems, including SEIG applications. In [21], a DO-based controller designed using Lyapunov’s method was implemented for a STATCOM-based SEIG system. Although DOs offer strong performance benefits, they are more complex than conventional methods and lack extensive validation under conditions of highly unbalanced and nonlinear loads, where voltage and frequency regulation remain difficult. A related Lyapunov function-based approach is also reported in [22]. More recently, [23] proposed a Lyapunov-based method using an Andronov–Hopf oscillator for load-current harmonic mitigation. Despite its technical strengths, [23] validates the approach only for balanced load current. In SEIG-based local energy systems, unbalanced loads are common and cannot be ignored. Therefore, the method could be improved by incorporating an orthogonal-signal generation technique. Note that although the Lyapunov-function-based approaches [21–23] offer a stronger stability guarantee than the local-linearization-based approach, in practice – due to measurement noise and other unmodeled disturbances - input-to-state stability is more appropriate in the context of power electronic system control.

In [24], the authors applied a leaky minimal disturbance theory (LMDT) neural network for load-current filtering, enabling high-performance SEIG operation under severely distorted loads. This technique adapts neural network weights in real time through an update law, but validation under measurement offsets and fault conditions remains limited. Comparable approaches include least mean square-based update laws [25] and Lorentzian norm-based update laws [26], mixed-step size normalized least means fourth update laws [27], adaptive normalized least mean absolute third [28], adaptive quantum normalized least-mean fourth [29], to name a few. A key limitation of these adaptive laws [24–29] is that they are nonlinear, and no explicit tuning laws are available for their gains. Therefore, trial-and-error methods are often used, which are sensitive to operating conditions. Additionally, the use of the signum function in [28] may cause chattering (high-frequency switching), as in classical first-order sliding-mode control where the signum term induces rapid switching.

To address the shortcomings of existing approaches – including limited harmonic robustness (inability to eliminate odd-order harmonics), lack of gain‑tuning formulas (leading to trial‑and‑error tuning), and reliance on offline optimization that fails to adapt to current operating conditions – this work introduces an extended enhanced phase-locked loop (E^2^PLL)-based control strategy for DSTATCOM operation. The main contribution of this study lies in the development of a control scheme that achieves a trade-off between structural complexity and filtering capability, thereby enabling precise estimation of load current magnitude and frequency. This allows the DSTATCOM to maintain balanced and sinusoidal source currents even under nonlinear and unbalanced loading conditions. The effectiveness of the proposed method is validated through comprehensive experimental studies, which demonstrate its practicality and robustness. The results confirm that the E^2^PLL-based control strategy provides a significant improvement in ensuring stable and reliable operation of wind energy systems.

Note that this article is an extended version of our preliminary work published in [30]. In this substantially expanded version, we provide full details of the control system, including modeling and tuning. In our study in [30], while regulating the voltage amplitude of the SEIG at the reference value under load changes, in this study we additionally ensure both the voltage amplitude and the frequency at their reference values. A comprehensive experimental comparison of the proposed PLL is realized. Besides, a real-time simulator-based comparative study of the DSTATCOM-supported SEIG system is presented.

In addition to the comparative evaluation with advanced PLL algorithms, this paper further includes several comparisons with recent DSTATCOM control strategies, highlighting the characteristics of the proposed controller in terms of harmonic rejection capability, dynamic response, computational complexity, parameter tuning, and experimental validation. Furthermore, additional robustness studies are carried out under practical operating disturbances, including wind-speed variations, measurement noise, and DC-link voltage ripple, to demonstrate the reliability of the proposed controller under realistic operating conditions.

The remainder of this article is organized as follows. First, an overview of the system architecture under consideration is presented. The proposed control strategy is then described in detail, including the development of the proposed PLL and a comparative analysis with related methods from the literature. This is followed by a real-time comparative study based on the considered system architecture. Finally, the main findings are summarized in the conclusions.

## DSTATCOM-based SEIG system

**Fig 1:**
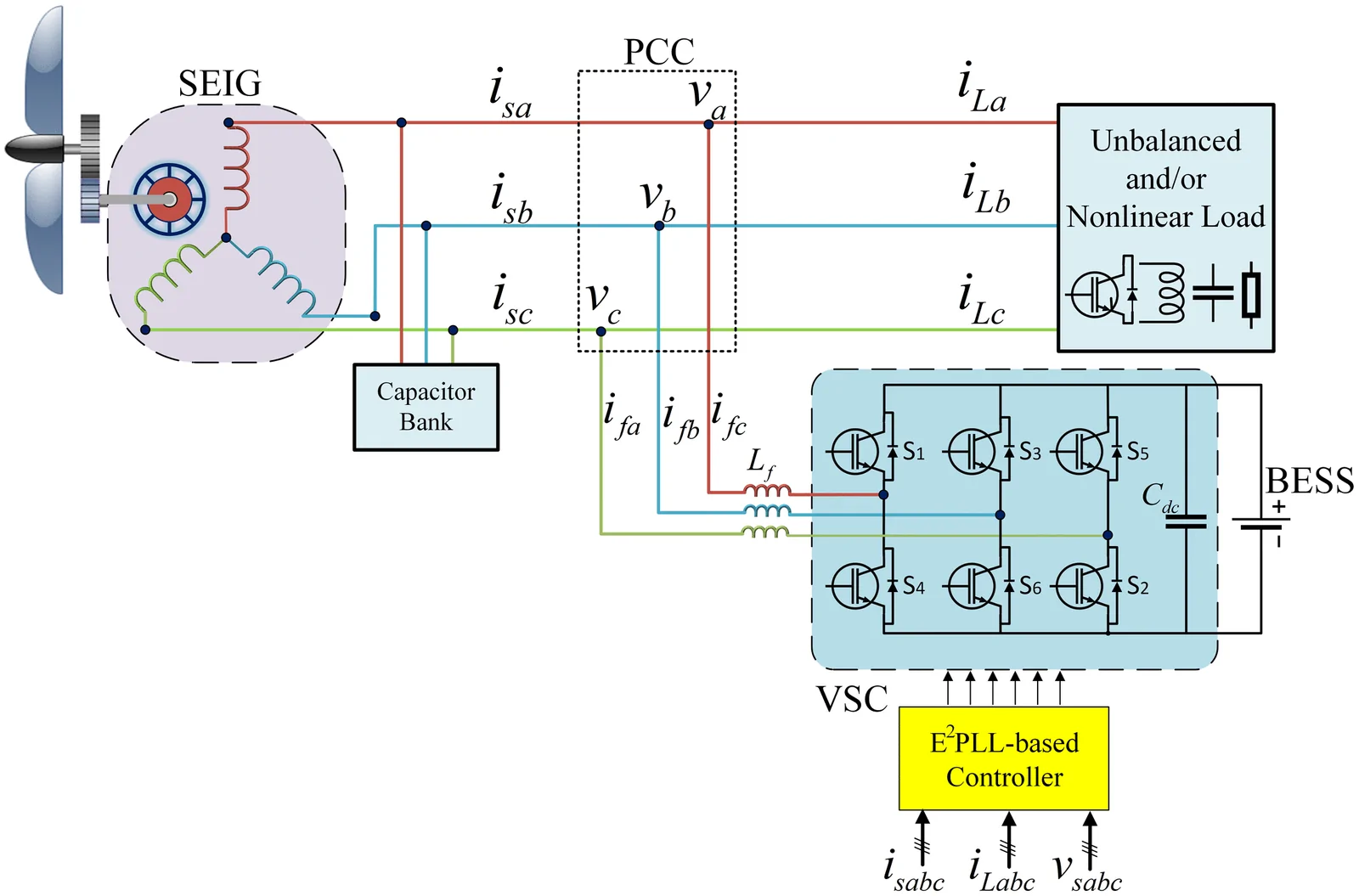
Block diagram of DSTATCOM-based SEIG system.

DSTATCOM-based SEIG system, which supplies the nonlinear and unbalanced loads frequently encountered in industry, is presented in Fig 1. SEIG is typically mechanically driven by a horizontal-axis wind turbine. A SEIG requires reactive power to generate voltage at the moment of initial operation [15]. At the start, this requirement is met by a group of capacitors connected to the generator terminals instead of the DSTATCOM. These capacitors contribute to the voltage formation process by providing the initial excitation [31].

The DSTATCOM connected in parallel to the point of common coupling (PCC) meets the additional reactive power demand required by both the load and the generator during load changes, while also compensating for unbalanced and harmonic distorting currents. The DSTATCOM structure consists of a voltage-source converter (VSC), a three-phase interconnection inductance ($L_{f}$) and a DC bus capacitor ($C_{dc}$). The IGBT-based switches in the VSC operate at a high switching frequency, providing fast dynamic compensation.

A battery energy storage system (BESS) integrated into the system supports the DC bus, contributing to stable operation. It also enhances the dynamic performance of the DSTATCOM by balancing power during sudden load changes and enables the SEIG to generate voltage at a nominal frequency of 50 Hz. The basic parameters of SEIG and DSTATCOM used in the system are given in Table 1. These parameters are used as a basis for mathematical modelling and simulation studies. Furthermore, the proposed structure is tested and validated on an Opal-RT-based hardware-in-the-loop (HIL) platform.

**Table 1:** Parameters of SEIG and DSTATCOM.

SEIG Parameters	
Power (P)	15 kW
Line voltage ($V_{\text{L-L}}$)	400 V
Rated current ($I_{\text{rated}}$)	28.79 A
Power factor (cosφ)	0.83
Stator resistance ($R_{\text{s}}$)	0.2147 Ω
Stator inductance ($L_{\text{s}}$)	0.000991H
Rotor resistance (${R^{\prime }}_{\text{r}}$)	0.2205 Ω
Rotor inductance (${L^{\prime }}_{\text{r}}$$L_{r}^{\prime }$)	0.000991 H
Mutual inductance ($L_{\text{m}}$)	0.06419 H
Frequency (f)	50 Hz
Pole pairs (p)	2
Excitation capacitor bank ($C_{\Delta }$)	65 μF (Δ-connected)
**DSTATCOM Parameters**	
Interfacing inductor ($L_{\text{f}}$)	5.85 mH
DC bus capacitor ($C_{\text{dc}}$)	2700 μF
Switching frequency ($f_{\text{s}}$)	10 kHz

## Proposed E^2^PLL-based control method for DSTATCOM

**Fig 2:**
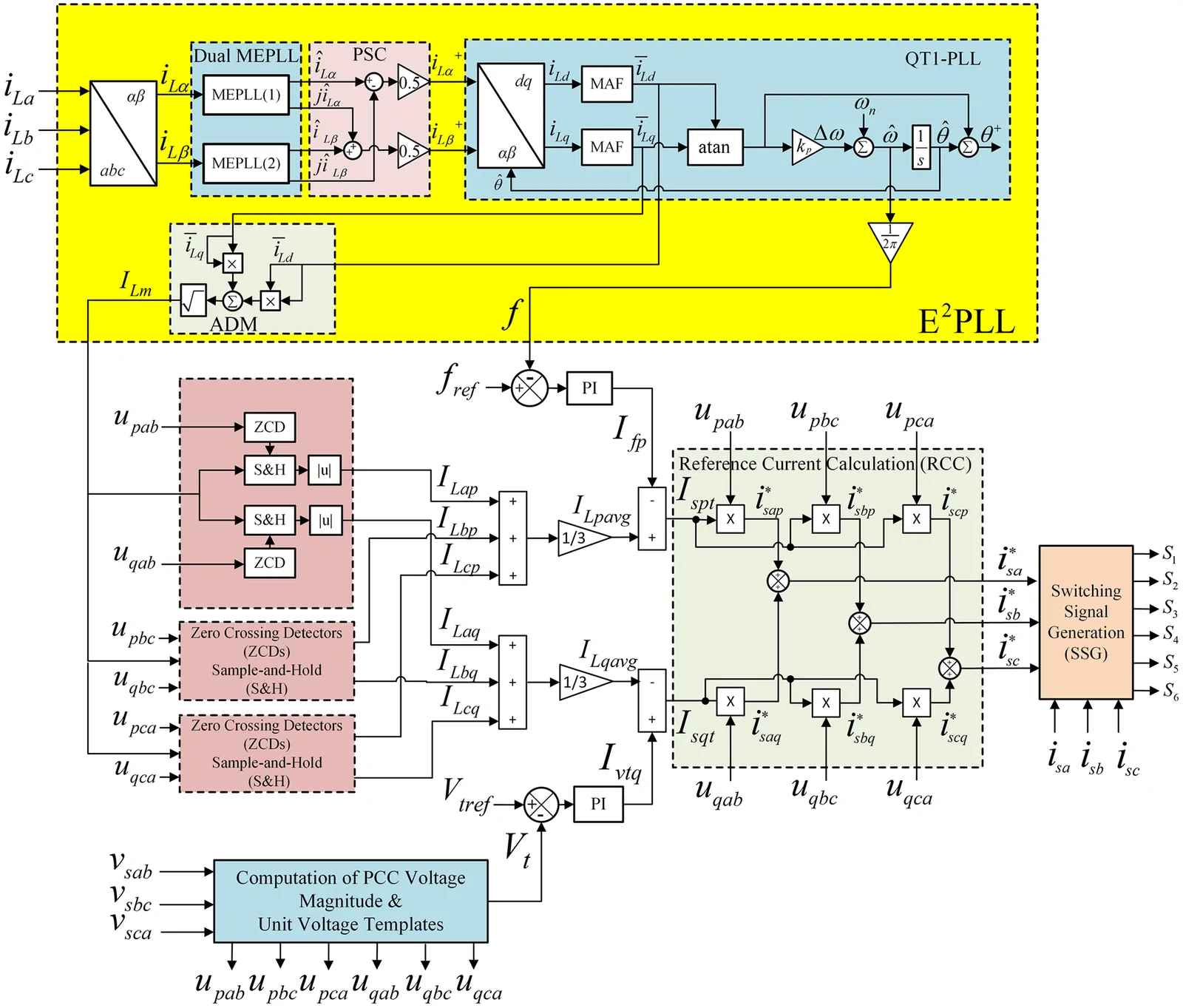
Proposed E^2^PLL-based control method for DSTATCOM.

Fig 2 shows the block structure of the proposed control algorithm for DSTATCOM. The proposed methodology employs the E^2^PLL technique to facilitate the precise estimation of the amplitude ($I_{Lm}$) and frequency (f) of load currents. The utilization of E^2^PLL with high disturbance cancellation capability ensures the accurate extraction of the fundamental active and reactive components of the load currents ($i_{Lp}$ and$i_{Lq}$), even in circumstances where unbalanced and nonlinear loads are connected to the PCC. Thus, in order to control the VSC of the DSTATCOM, the reference currents ($i_{sa}^{*}$, $i_{sb}^{*}$, and$i_{sc}^{*}$) are determined appropriately.

The proposed E^2^PLL method for DSTATCOM control will be described in detail in the next subsection.

### Description of proposed E^2^PLL

**Fig 3:**
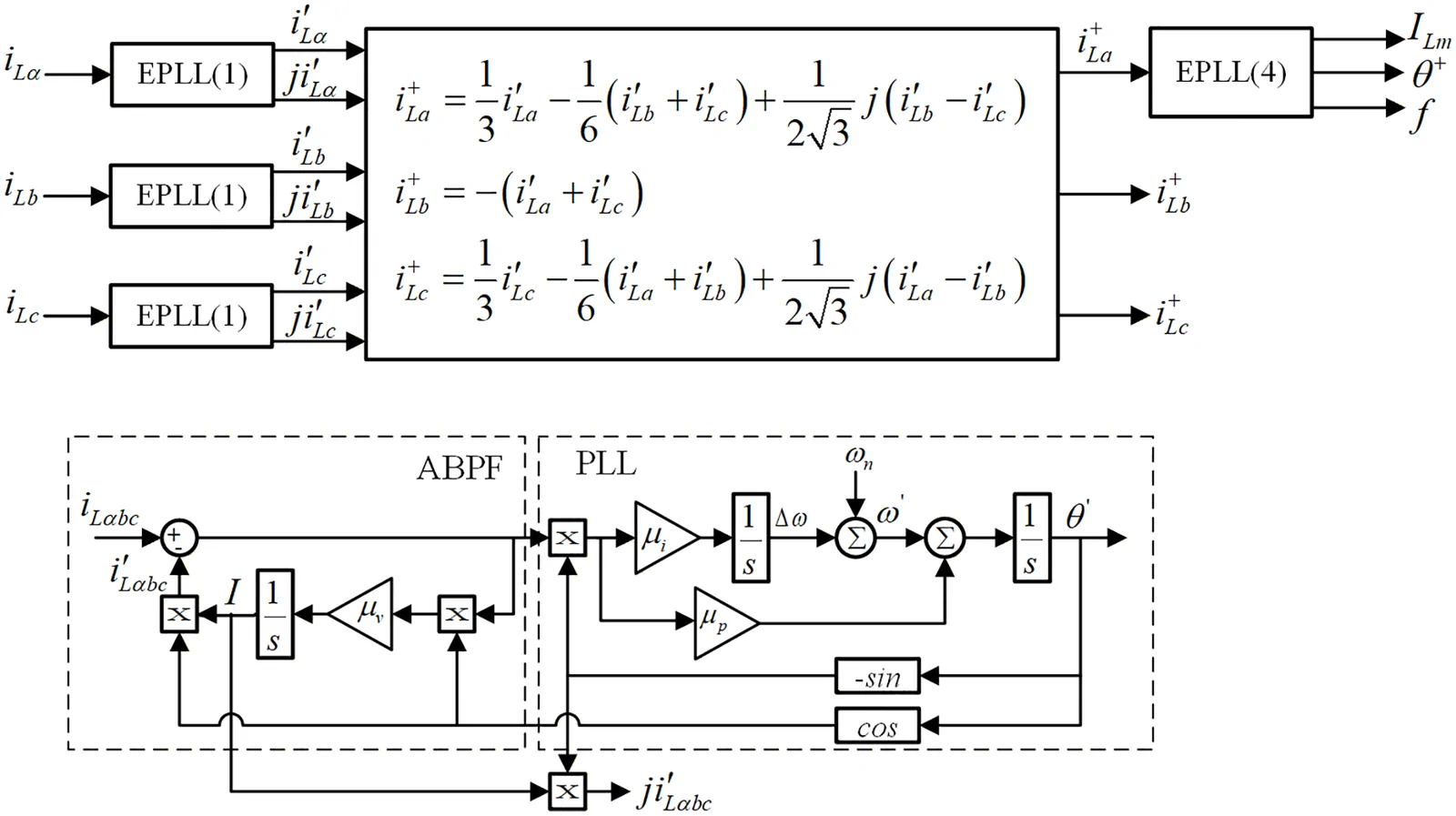
(a) Block diagram of the standard three-phase EPLL; (b) Single-phase EPLL structure, where (a) is top and (b) is bottom.

**Fig 4:**
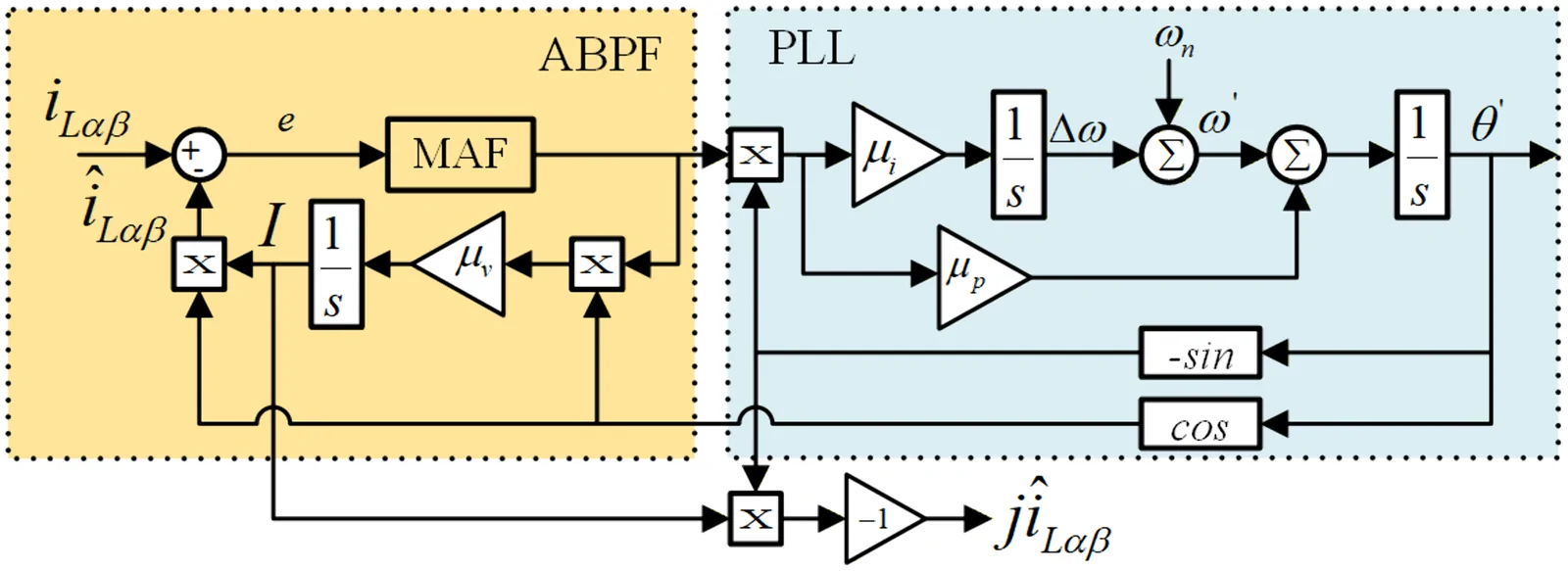
MEPLL structure.

The block diagram of the proposed E^2^PLL is depicted in Fig 2. The E^2^PLL proposed by Sevilmiş and Karaca in [32] is an improved version of the standard three-phase EPLL. In the standard three-phase EPLL [33], as shown in Fig 3(a), four single-phase EPLL structures are required to estimate the amplitude, phase, and frequency of the input signals. Fig 3(b) depicts the block diagram of single-phase EPLL. Although the standard three-phase EPLL has good filtering capabilities, particularly under unbalanced conditions, it is structurally complex and involves a high computational load, which makes its real-time implementation difficult. The proposed E^2^PLL architecture, however, has been optimized to include only two modified EPLL (MEPLL) units in order to overcome this limitation of the standard EPLL method. The block structure of a MEPLL is shown in Fig 4. Each MEPLL comprises an adaptive band-pass filter (ABPF) and a PLL. It also has a moving average filter (MAF) in the ABPF. The employment of the MAF situated along the error signal path serves to enhance the filtering capability of the MEPLL. Consequently, decreasing the quantity of single-phase EPLLs from four to two contributes to a reduction in the structural complexity of the system while providing significantly stronger harmonic filtering capabilities thanks to the ABPF and MAF integrated into the MEPLL. This makes it possible to estimate the amplitude, phase, and frequency of the load currents much more accurately under nonlinear and unbalanced load conditions.

As illustrated in Fig 4, the load currents in αβ -frame ($i_{L\alpha }$ and $i_{L\beta }$) are passed through the ABPF structure, and their filtered forms (${\hat{i}}_{L\alpha }$ and ${\hat{i}}_{L\beta }$) and quadrature forms ($j{\hat{i}}_{L\alpha }$ and$j{\hat{i}}_{L\beta }$) are obtained. These currents can be expressed as


$$\begin{array}{c} \begin{array}{c} i_{L\alpha }=I_{Lm}\text{\textbackslash{}hspace\{0.17em}\}cos\left(\theta \right);\text{\textbackslash{}hspace\{0.17em}\;\}i_{L\beta }=I_{Lm}\text{\textbackslash{}hspace\{0.17em}\}sin\left(\theta \right)\\ {\hat{i}}_{L\alpha }=I\text{\textbackslash{}hspace\{0.17em}\}cos\left(\theta^{\prime }\right);\text{\textbackslash{}hspace\{0.17em}\;\;\;\;\}{\hat{i}}_{L\beta }=I\text{\textbackslash{}hspace\{0.17em}\}sin\left(\theta^{\prime }\right)\\ j{\hat{i}}_{L\alpha }=I\text{\textbackslash{}hspace\{0.17em}\}sin\left(\theta^{\prime }\right);\text{\textbackslash{}hspace\{0.17em}\;\;\}j{\hat{i}}_{L\beta }=-Icos\left(\theta^{\prime }\right) \end{array} \end{array}$$ (1)

where $I_{Lm}$ and θ denote the amplitude and phase angle of the load currents, respectively. I and $\theta^{\prime }$ represent the amplitude and phase angle of the filtered load currents, respectively. It is assumed that $\theta^{\prime }\approx \theta$ and $I\approx I_{Lm}$ in a quasi-locked condition.

In ABPF, the error signal (e) from the MAF is fed into a PLL as an input, and the PLL estimates the frequency and phase of the load currents. The window-length of MAF is designated as *T*/8 (in which *T* denotes the fundamental grid period) with the objective of enhancing the system’s stability [32]. It should be noted that the MEPLL has its own frequency estimation mechanism thanks to the PLL in its structure. In this way, it maintains high filtering performance even during frequency changes.

The MEPLL includes three gains to control its dynamics. $\mu_{v}$ in ABPF controls the convergence speed of I. A larger $\mu_{v}$ reduces the convergence time of I; however, it causes oscillations. $\mu_{p}$ and $\mu_{i}$ in the PLL control loop are the PI parameters that adjust the PLL dynamics.

The differential equations of the MEPLL are determined based on the cost function J [32] expressed as follows:


$$\begin{array}{c} J=\frac{\text{1}}{\text{2}}e^{\text{2}}=\frac{\text{1}}{\text{2}}{\left(i_{L\alpha \beta }-{\hat{i}}_{L\alpha \beta }\right)}^{\text{2}} \end{array}$$ (2)

Based on the gradient descent method, the equations are obtained as


$$\begin{array}{c} \begin{array}{c} \dot{I}=\mu_{v}ecos\left(\theta^{\prime }\right)\\ \Delta \dot{\omega }=-\mu_{i}esin\left(\theta^{\prime }\right)\\ \dot{\theta }=\omega_{n}+\Delta \omega -\mu_{p}esin\left(\theta^{\prime }\right) \end{array} \end{array}$$ (3)

where $\omega_{n}$ and Δω are the fundamental frequency and the deviation from the fundamental frequency, respectively.

Based on the linear analysis of the MEPLL, the characteristic equations of amplitude and frequency/phase estimation loops can be derived as [32]


$$\begin{array}{c} \begin{array}{c} \text{\textbackslash{}hspace\{0.17em}\;\;\;\;\;\;\;\;\;\;\;\;\;\;\;\;\;\;\;\;\;\;\}s+\frac{\mu_{v}}{\text{2}}\text{MAF}\left(s\right)=0\\ s^{\text{2}}+\frac{\mu_{p}I_{Lm}}{\text{2}}\text{MAF}\left(s\right)s+\frac{\mu_{i}I_{Lm}}{\text{2}}\text{MAF}\left(s\right)=0 \end{array} \end{array}$$ (4)

It can be seen from (4) that the characteristic equation of the frequency/phase estimation loop is similar to that of a standard PLL and is identical to it when MAF(s) = 1. In other words, both MEPLL and standard PLL are based on the same linear model.

As illustrated in Fig 2, the filtered load currents at MEPLL outputs (i.e., ${\hat{i}}_{L\alpha }$,$j{\hat{i}}_{L\alpha }$,${\hat{i}}_{L\beta }$, and$j{\hat{i}}_{L\beta }$) are transferred to the positive sequence calculator (PSC) unit expressed in (5) to detect the positive sequence components of load currents ($i_{L\alpha }^{+}$ and$i_{L\beta }^{+}$).


$$\begin{array}{c} \left[\begin{array}{c} @c@i_{L\alpha }^{+}\\ i_{L\beta }^{+} \end{array}\right]=\frac{\text{1}}{\text{2}}\left[\begin{array}{cc} @cc@1 & -j\\ j & 1 \end{array}\right]\left[\begin{array}{c} @c@{\hat{i}}_{L\alpha }\\ {\hat{i}}_{L\beta } \end{array}\right] \end{array}$$ (5)

The positive components of the load currents $i_{L\alpha }^{+}$ and$i_{L\beta }^{+}$ are then given as inputs to a quasi-type-1 PLL (QT1-PLL) [34] to obtain the amplitude ($I_{Lm}$) and frequency (f) of load currents. In contrast to the PI controller employed in typical control loops, the QT1-PLL uses a proportional gain ($k_{p}$) within its control loop. It appears to be a type-1 system, but it is a type-2 system actually due to forward path to the phase angle output. Thus, the E^2^PLL follows the phase and frequency jumps without steady-state error.

**Fig 5:**
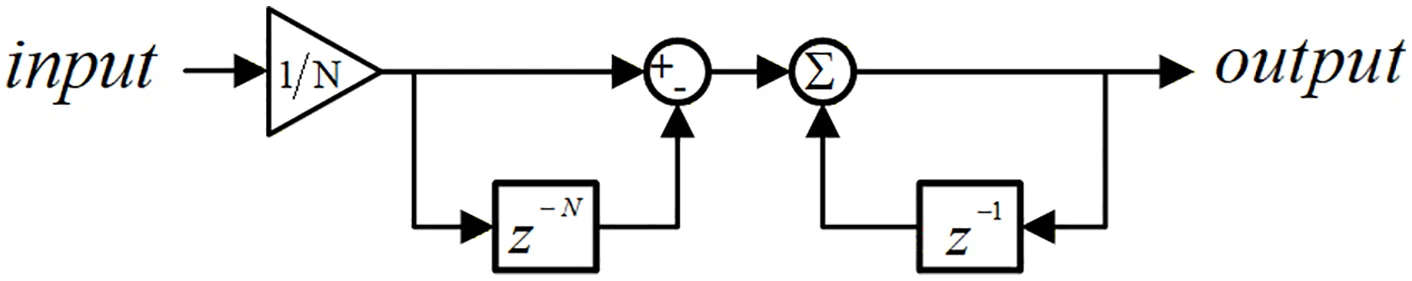
Block structure of MAF.

A moving average filter (MAF) in the control loop of QT1-PLL enhances the filtering capability of the E^2^PLL. The MAF can behave as an ideal low-pass filter under specific conditions. Its continuous-time and discrete-time transfer functions can be obtained as in (6) and (7), respectively [35].


$$\begin{array}{c} \text{MAF}\left(s\right)=\frac{1-e^{-T_{\omega }s}}{T_{\omega }s} \end{array}$$ (6)

$$\begin{array}{c} \text{MAF}\left(z\right)=\frac{\text{1}}{N}\frac{1-z^{-N}}{1-z^{-1}} \end{array}$$ (7)

where $T_{\omega }$ is window-length of MAF and MAF’s order N is equal to $f_{s}T_{\omega }$, where $f_{s}$ denotes the sampling frequency. It is possible to obtain the block diagram of MAF using (7), as demonstrated in Fig 5. As can be seen, the MAF structure is uncomplicated and easy to incorporate into digital applications by determining the value of N [36]. In this study, N is calculated as 33 by taking $f_{s}=5\text{\textbackslash{}hspace\{0.5em}\;kHz\}$ and $T_{\omega }=T/3$, where T is fundamental period of load currents.

The amplitude of load currents ($I_{Lm}$) is computed from the amplitude detection mechanism (ADM) unit, as can be seen in Fig 2. In this way, accurate detection of the amplitude in frequency drifts is ensured. The ADM is given by:


$$\begin{array}{c} I_{Lm}=\sqrt{{\overset{―}{i}}_{Ld}^{\text{2}}+{\overset{―}{i}}_{Lq}^{\text{2}}} \end{array}$$ (8)

### Parameter design procedure of proposed E^2^PLL

**Fig 6:**
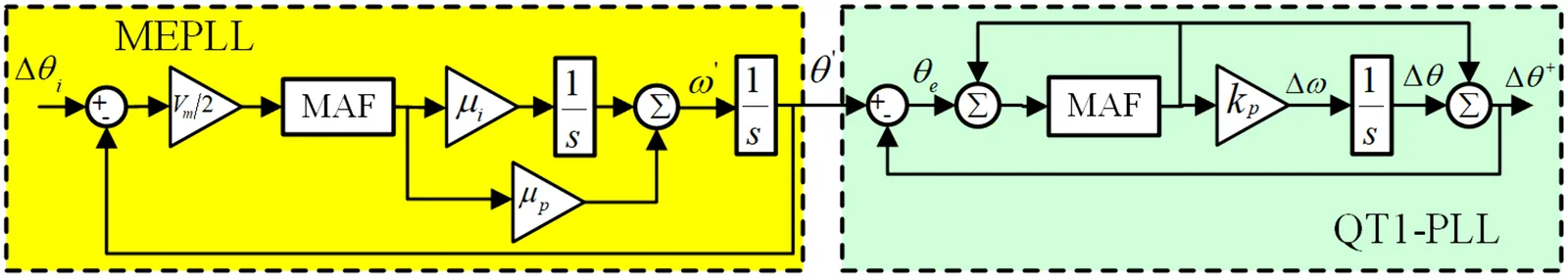
Linear model of proposed E^2^PLL.

We presented the detailed information about the parameter design of the E^2^PLL in [32]. However, the key tuning procedures and design guidelines are summarized here to improve reproducibility. In order to configure the control parameters of E^2^PLL, its linear model is considered, as depicted in Fig 6. It has previously been determined that the $T_{\omega }$ value of the MEPLL is T/8 and the $T_{\omega }$ value of the MAF in the QT1-PLL is T/3; this indicates that only one parameter (i.e., $k_{p}$) remains to be designed. Note that the control parameters of the MEPLL ($\mu_{v}$, $\mu_{p}$, and $\mu_{i}$) are set to the same values as those of the standard single-phase EPLL shown in Fig 3(b). That is, $\mu_{v}=\mu_{p}=444$ and $\mu_{i}=50000$ [33].

Based on the linear model in Fig 6, the open-loop transfer function in *s*-domain can be obtained as:


$$\begin{array}{c} G_{ol}\left(s\right)=\frac{\Delta \theta^{+}\left(s\right)}{\theta_{e}}=\left(\frac{\text{MAF}\left(s\right)}{1-\text{MAF}\left(s\right)}\right)\left(1+\frac{k_{p}}{s}\right) \end{array}$$ (9)

Using (9) and Fig 6, the transfer function of phase-tracking-error can be expressed as:


$$\begin{array}{c} G_{e}\left(s\right)=\frac{\theta_{e}}{\Delta \theta_{i}\left(s\right)}=\underset{\text{MEPLL}\left(s\right)}{\underbrace{\frac{\left(\mu_{p}V_{m}/2\right)\text{MAF}\left(s\right)s+\left(\mu_{i}V_{m}/2\right)\text{MAF}\left(s\right)}{s^{\text{2}}+\left(\mu_{p}V_{m}/2\right)\text{MAF}\left(s\right)s+\left(\mu_{i}V_{m}/2\right)\text{MAF}\left(s\right)}}}\left(\frac{\text{1}}{1+G_{ol}\left(s\right)}\right) \end{array}$$ (10)

**Fig 7:**
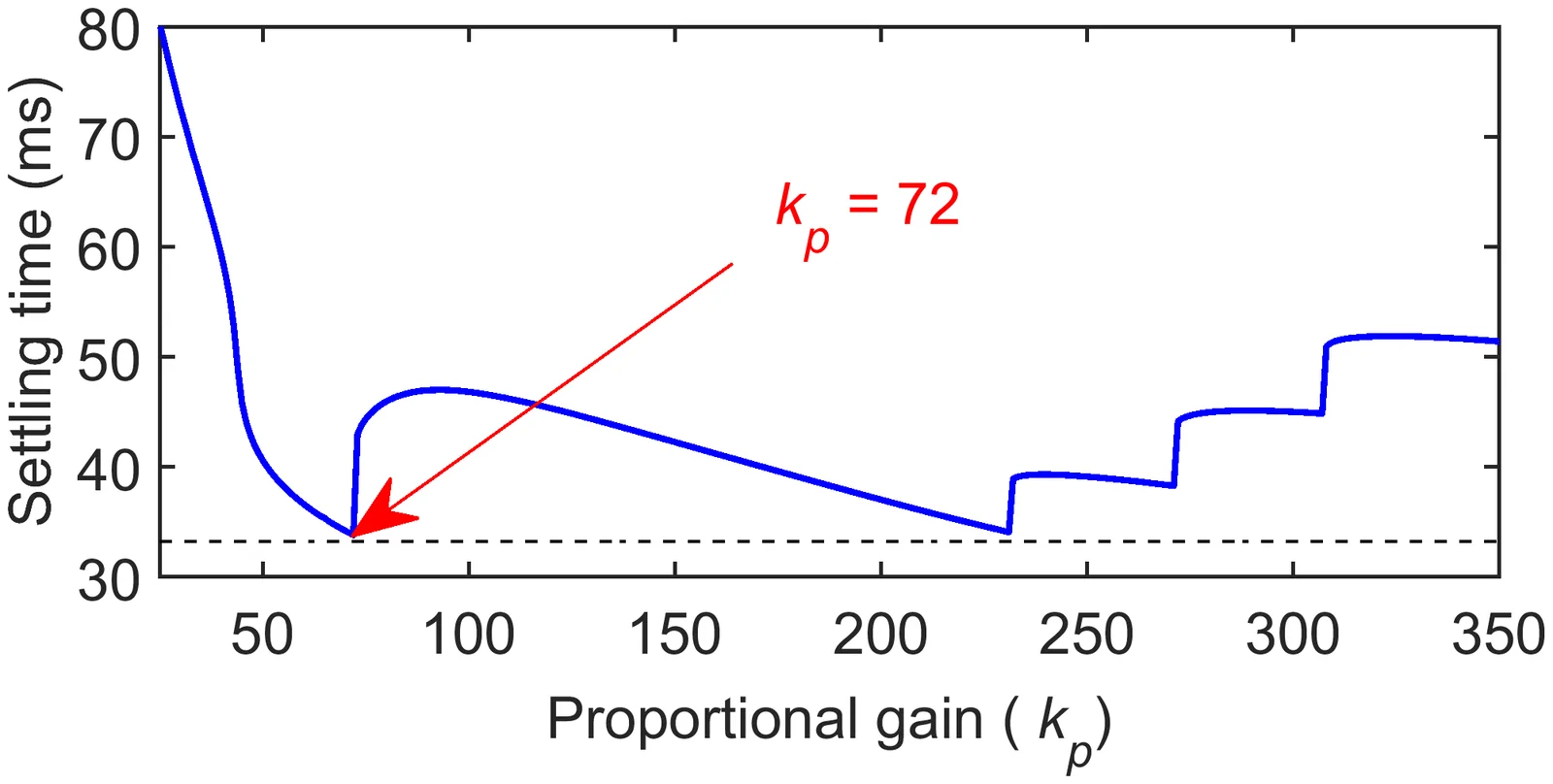
2% settling-time of E^2^PLL under phase jump as a function of $k_{p}$.

From (10), the change of the 2% settling-time of the E^2^PLL as a function of $k_{p}$ under a phase jump condition is obtained, as illustrated in Fig 7. It is determined that the $k_{p}$ of the E^2^PLL is set to 72 in order to achieve the minimum settling-time.

### Stability analysis of proposed E^2^PLL

**Fig 8:**
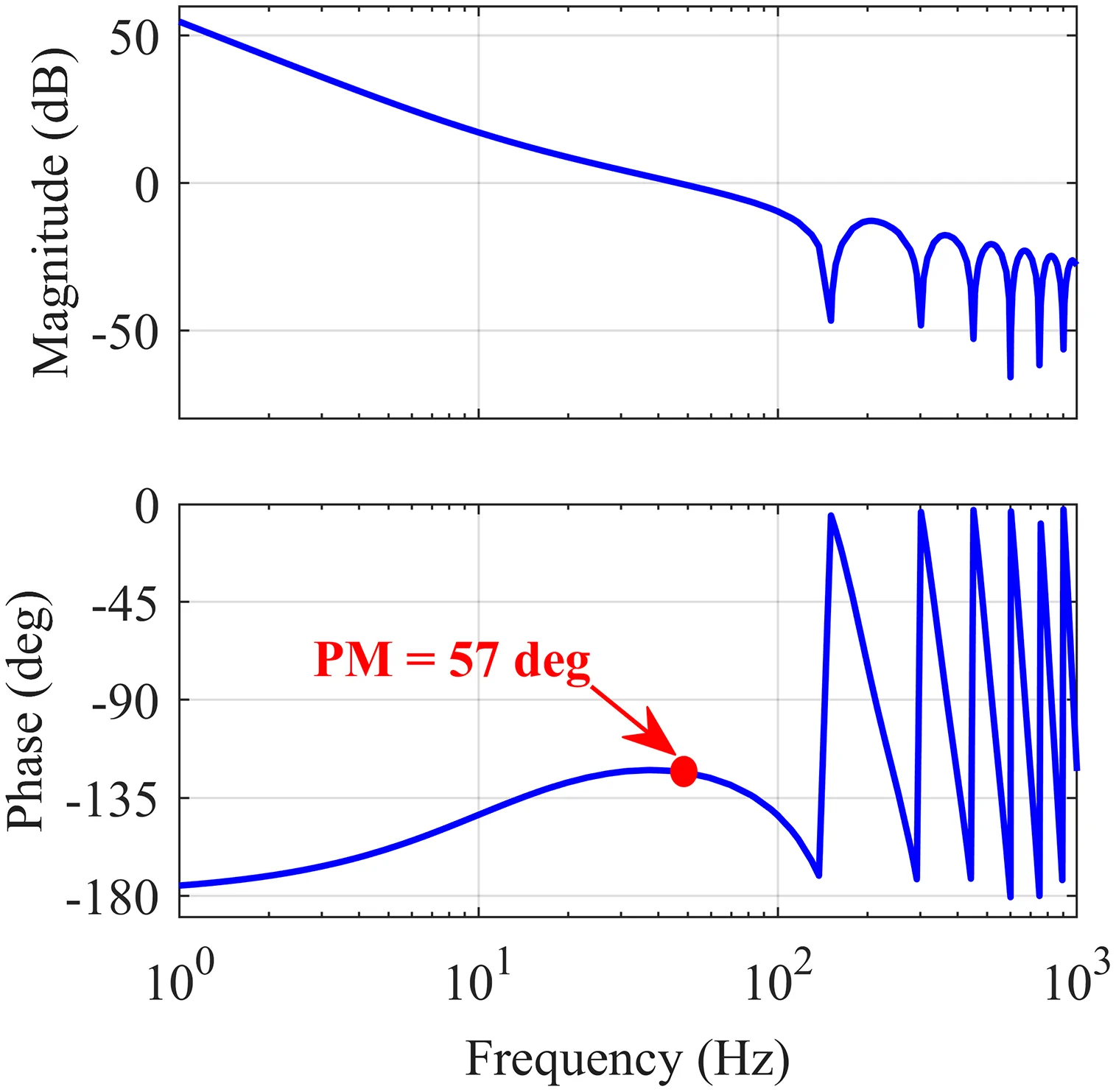
Bode diagram of proposed E^2^PLL.

To evaluate the stability of proposed E^2^PLL, its open-loop transfer function in (10) is used [32]. The open-loop bode diagram of the E^2^PLL is derived using (10), as illustrated in Fig 8. As shown, its phase margin (PM) is 57°, which is within the recommended range of 30° – 60° [37]. Consequently, this figure verifies that the E^2^PLL ensures the stability of the system.

### Performance comparison of advanced PLLs

**Fig 9:**
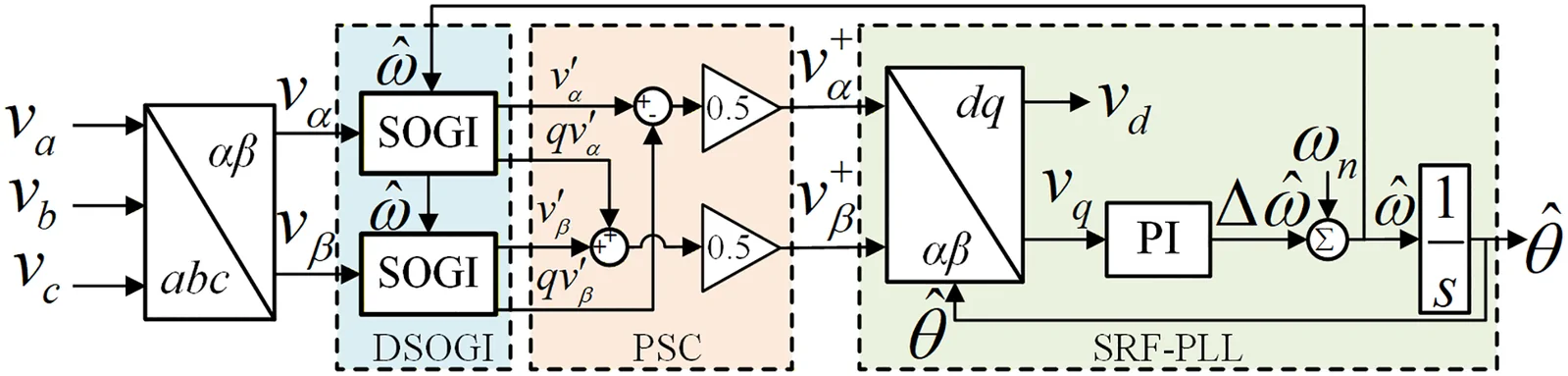
Block diagram of DSOGI-PLL.

**Fig 10:**
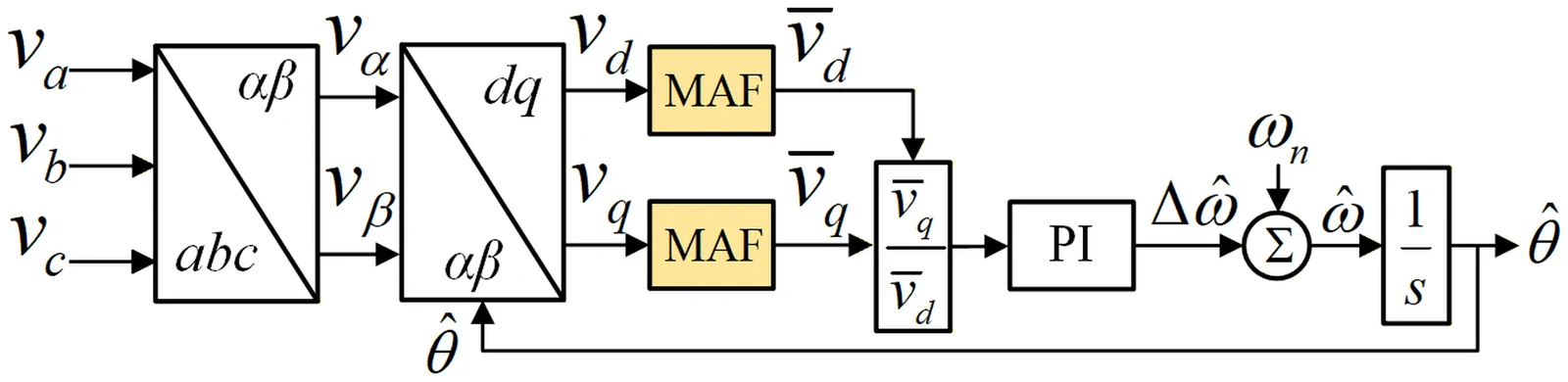
Block diagram of MAF-PLL.

In this section, the performance of the proposed E^2^PLL is demonstrated through various experimental studies. Besides, its performance is compared with two advanced and well-known PLL methods. These are DSOGI-PLL [38] and MAF-PLL [35]. Their block diagrams are shown in Figs 9 and 10, respectively. To improve the readability of the article, the details of these two PLL methods are not provided.

In experiments using a TMS320F28335 DSP, the sampling frequency is 5 kHz, and the amplitudes of the load currents are assumed to be 1 p.u. The control parameters used in PLLs are summarized in Table 2.

**Table 2:** Control parameters of PLLs.

Parameters	DSOGI-PLL [38]	MAF-PLL [35]	Proposed E^2^PLL [32]
Proportional gain,$k_{p}$	138.23	83.33	72
Integral gain, $k_{i}$	7961	2893.5	–
SOGI’s damping factor, k	2.11	–	–
MAF’s window-length,$T_{\omega }$	–	*T/*2	*T*/3
MAF’s order,N	–	50	33

Three tests are considered as follows:

***Test-1***: Imbalances occur in load currents. The amplitudes of phase-A, phase-B, and phase-C are 1 p.u., 1.2 p.u., and 0.8 p.u., respectively. Most PLLs in the literature that overcome imbalances incorrectly estimate the amplitude, phase, and frequency of the input signal when there is both imbalances and frequency jump. Therefore, in this test, the frequency jumps from 50 to 52 Hz under unbalanced conditions.

***Test-2***: The fifth and seventh odd-order harmonics in load currents are created, where $V_{\text{5}}^{-}=0.04∠-90^{\circ }$ and $V_{\text{7}}^{+}=0.04∠0^{\circ }$, in accordance with IEEE Std. 1547–2018 [39]. The frequency also changes from 50 Hz to 47 Hz in this test. Also, to provide a more dynamic harmonic profile, inter-harmonics at 4.1f (192.7 Hz) and 5.8f (272.6 Hz) are added into the load currents, where f is 47 Hz. The total harmonic distortion (THD) of the load currents in this test is about 6.74%.

***Test-3:*** Single-phase open-circuit fault occurs. That is, the current drawn from one of the phases is zero. The frequency is set to 50 Hz.

**Fig 11:**
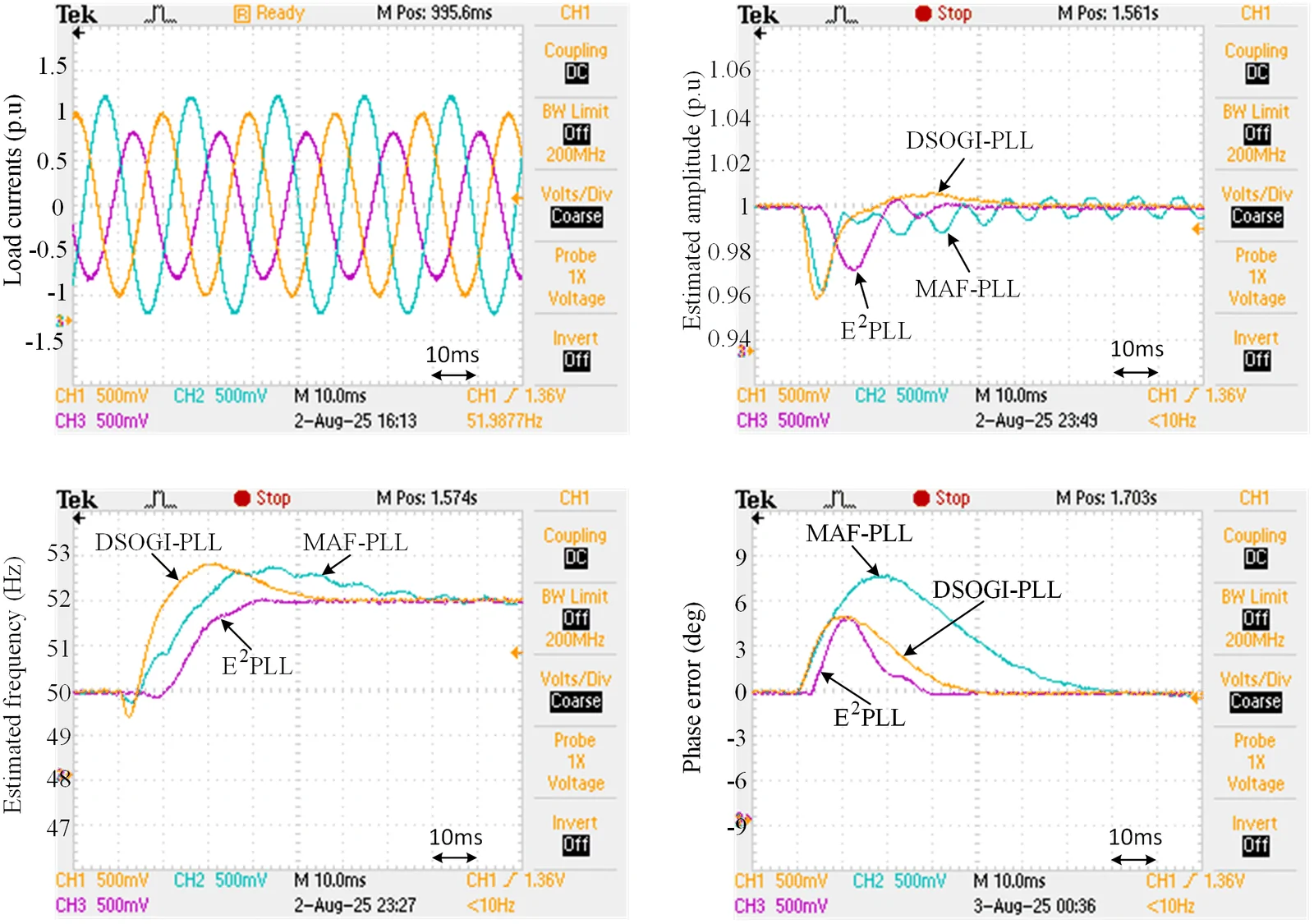
Test-1: Unbalanced load condition with frequency jumps. (a) Load currents; (b) Estimated amplitude; (c) Estimated frequency; (d) Phase error, where (a) is top-left, (b) is top-right, (c) is bottom-left, and (d) is bottom-right.

Fig 11 demonstrates the experimental results of Test-1 under unbalanced conditions with frequency deviation. As shown, the proposed E^2^PLL presents a faster transient response than the DSOGI-PLL and MAF-PLL. The settling-time of proposed method is 26 ms, while this time is 42.6 ms in DSOGI-PLL. Note that the MAF-PLL cannot settle according to the settling time of 2%. Additionally, the E^2^PLL provides lower amplitude and frequency overshoots than the DSOGI-PLL and MAF-PLL. Detailed numerical results are also given in Table 3. As can be seen in Fig 11, neither the DSOGI-PLL nor the E^2^PLL show any steady-state errors in amplitude, frequency and phase due to their frequency adaptation mechanisms. However, the MAF-PLL encounters double frequency oscillation errors when estimating the input signal information due to the frequency variations, and this can negatively affect the overall performance of the system. As a result, the proposed E^2^PLL provides perfect imbalance rejection capability and superior dynamic performance under unbalanced conditions, even in the presence of frequency drifts.

**Table 3:** Experimental results of PLL methods.

	DSOGI-PLL [38]	MAF-PLL [35]	Proposed E^2^PLL [32]
**Test-1: Unbalanced condition with frequency jumps**			
2% settling time	42.6 ms (2.3 cycles)	–	26 ms (1.3 cycles)
Amplitude overshoot	0.042 p.u.	0.036 p.u.	0.028 p.u.
Frequency overshoot	0.75 Hz	0.74 Hz	≈0 Hz
Phase overshoot	4.8°	7.4°	4.8°
**Test-2: Harmonic conditions with frequency jumps**			
2% settling time	–	–	23 ms (1.15 cycles)
Amplitude overshoot	0.036 p.u.	0.029 p.u.	0.006 p.u.
Frequency overshoot	1.5 Hz	1.1 Hz	≈0 Hz
Phase overshoot	7.7°	11.4°	7°
**Test-3: Single-phase open-circuit fault**			
2% settling time	≈100 ms (5 cycles)	≈40 ms (2 cycles)	≈30 ms (1.5 cycles)
Amplitude overshoot	0.1 p.u.	0.01 p.u.	0.0038 p.u.
Frequency overshoot	5.7 Hz	3.1 Hz	1.2 Hz
Phase overshoot	15°	5.5°	7.7°

**Fig 12:**
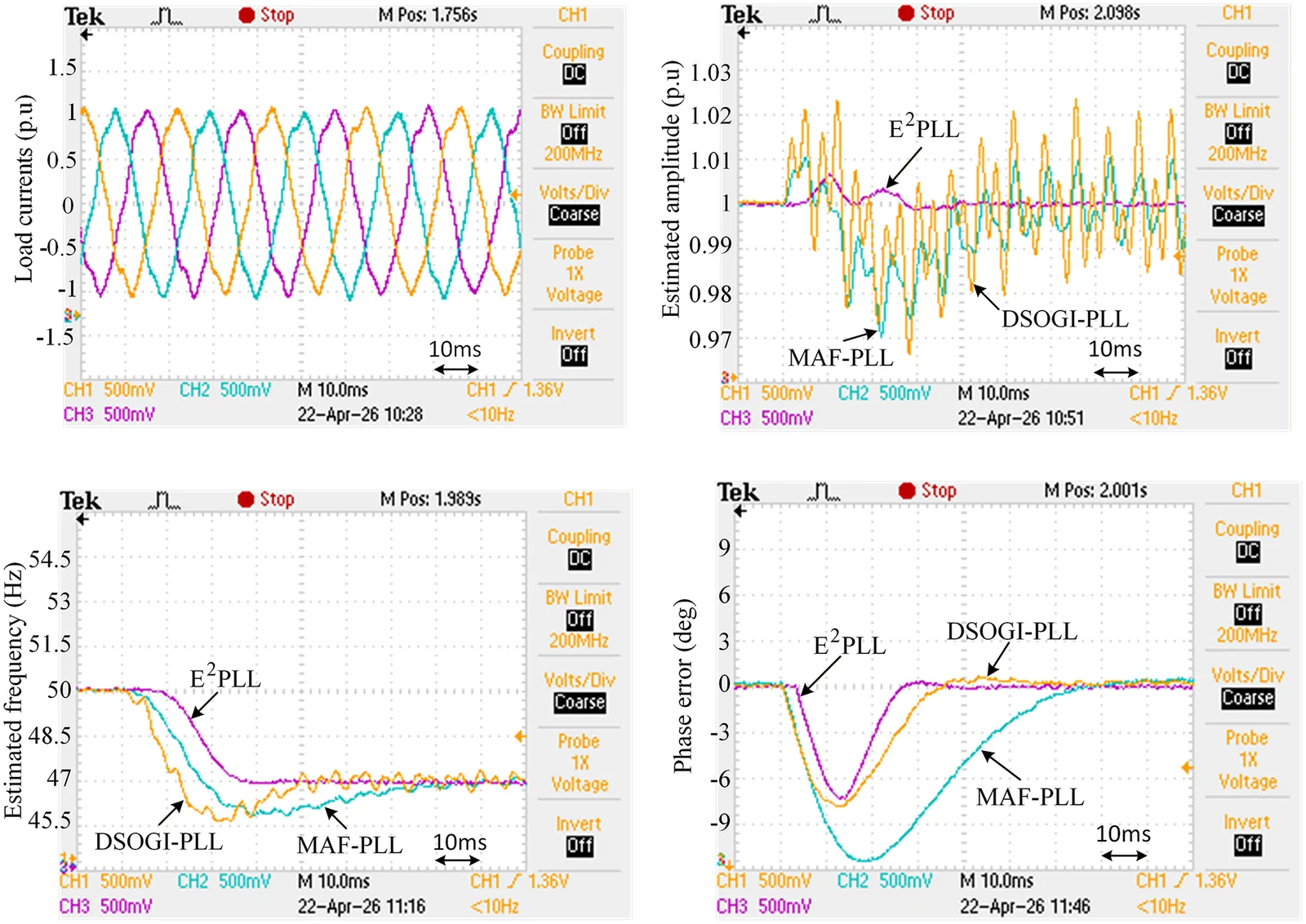
Test-2: Nonlinear load condition with frequency jumps. (a) Load currents; (b) Estimated amplitude; (c) Estimated frequency; (d) Phase error, where (a) is top-left, (b) is top-right, (c) is bottom-left, and (d) is bottom-right.

Fig 12 shows the experimental results of Test-2 under harmonic conditions with frequency deviation. As observed, the proposed PLL is superior to the other two methods in terms of dynamic response, accuracy, and overshoot. The DSOGI-PLL causes significant errors, particularly in amplitude and frequency estimation, which makes it difficult to use in the DSTATCOM applications. On the other hand, the MAF-PLL provides moderate performance in terms of steady-state accuracy; moreover, it offers a slower dynamic performance than the E^2^PLL. It cannot settle according to the settling time of 2%, while the settling-time of E^2^PLL is about 23 ms. The numerical results of Test-2 are presented in detail in Table 3. Consequently, this test confirms that the proposed E^2^PLL method performs better than DSOGI-PLL and MAF-PLL methods under harmonic conditions.

**Fig 13:**
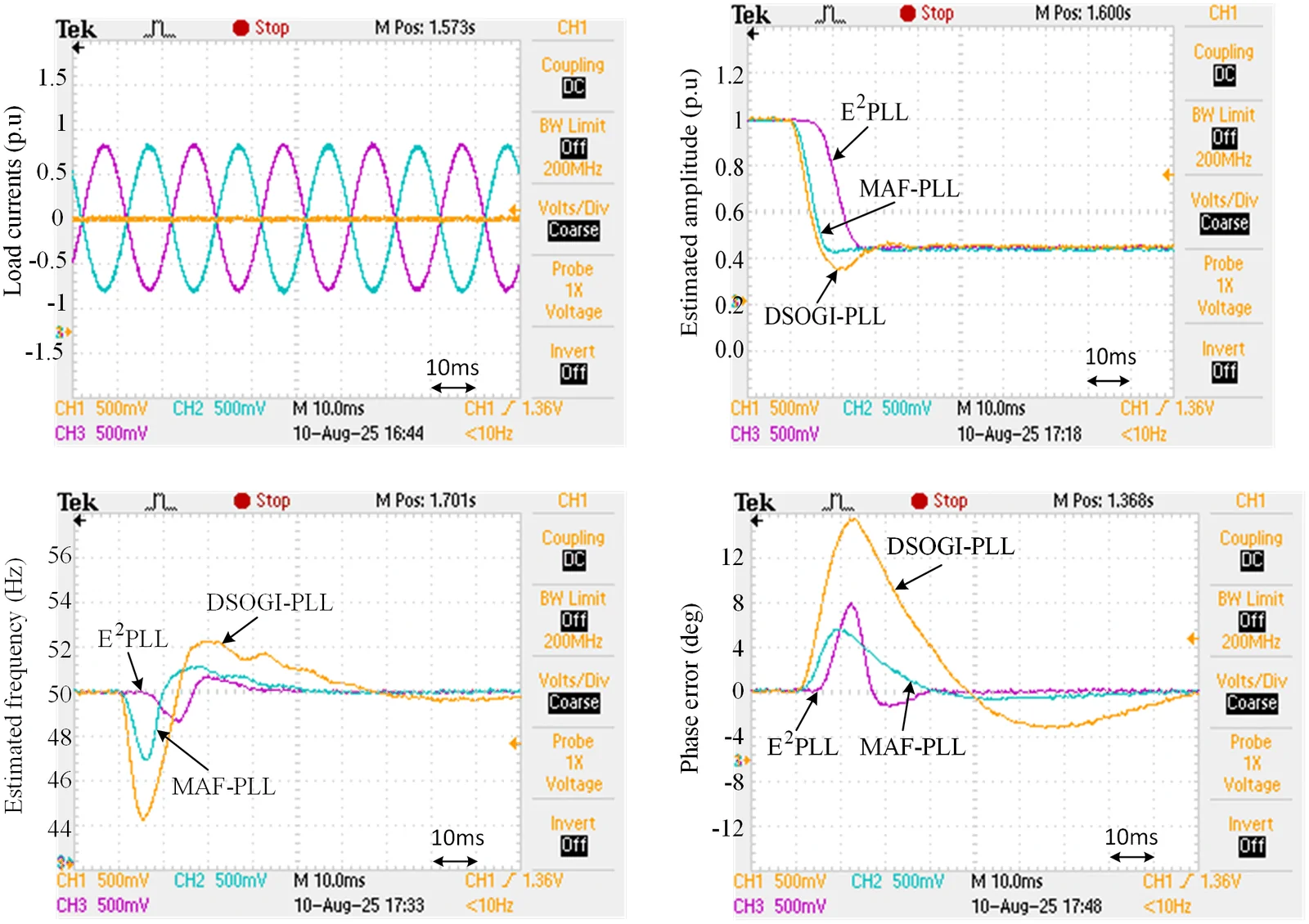
Test-3: Single-phase open-circuit fault. (a) Load currents; (b) Estimated amplitude; (c) Estimated frequency; (d) Phase error, where (a) is top-left, (b) is top-right, (c) is bottom-left, and (d) is bottom-right.

Fig 13 illustrates the experimental results of Test-3 in case of single-phase open-circuit fault. As can be seen, all PLLs accurately detect the amplitude, frequency, and phase of the input signal in steady-state. However, as shown in Fig 13 and Table 3, the DSOGI-PLL exhibits the slowest transient response and causes the largest overshoots compared to the MAF-PLL and E^2^PLL. Its settling time is about 100 ms, while the settling-times of MAF-PLL and E^2^PLL are about 40 ms and 30 ms, respectively. As shown in Table 3, the E^2^PLL provides lower overshoots than the MAF-PLL, except for phase overshoot. As a result, the proposed E^2^PLL provides a suitable solution for DSTATCOM control by offering both superior dynamic performance and precise steady-state response.

The comparative results presented in Table 3 are quite useful for evaluating the advantages and limitations of the DSOGI-PLL, MAF-PLL, and proposed E^2^PLL methods. It is important to note that no PLL method can be considered optimal under all operating conditions. The results obtained under three different challenging test conditions can be summarized as follows.

Firstly, the DSOGI-PLL provides effective positive and negative sequence separation capability under unbalanced conditions; however, as observed in Fig 12 and Table 3, significant errors occur in amplitude and frequency estimation under harmonic conditions. This limitation mainly arises from the restricted filtering capability of the DSOGI-PLL structure. Furthermore, as shown in Fig 13, its dynamic response deteriorates significantly in case of single-phase open-circuit fault.

On the other hand, the MAF-PLL demonstrates strong harmonic suppression performance thanks to the filtering capability of the MAF in its structure. As indicated by the results in Table 3, MAF-PLL provides acceptable accuracy under steady-state. Nevertheless, as shown in Fig 11, when the frequency deviates from the fundamental value of 50 Hz, the MAF-PLL causes fluctuations, particularly in amplitude and frequency estimation. Moreover, its dynamic response becomes considerably slower due to the inherent delay introduced by its filtering mechanism, and it causes large overshoots in phase estimation.

In contrast, the proposed E^2^PLL combines adaptive filtering and frequency tracking capabilities, providing superior performance under both harmonic and unbalanced conditions. As clearly demonstrated in Table 3, the proposed method achieves lower settling time, reduced overshoot, and higher estimation accuracy. However, the use of multiple filtering stages slightly increases computational complexity compared to two other PLL structures.

In conclusion, the proposed E^2^PLL method offers a well-balanced solution in terms of dynamic performance, filtering capability, and robustness against disturbances, and presents a strong alternative to existing methods in the literature.

### Comparative analysis of proposed E^2^PLL with existing methods

Table 4 presents a comparative evaluation of the proposed E²PLL and five PLL algorithms reported in the literature. These benchmark algorithms consist of the EPLL [33], DSOGI-PLL [38], MAF-PLL [35], TPSF-EPLL [40], I-αβ -EPLL [41]. This comparison takes into account the number of mathematical operators, the number of trigonometric functions, the number of integrators, memory usage, execution time, and filtering capability. As can be seen, while the proposed E²PLL requires nearly the same number of mathematical operators as the standard three-phase EPLL, it reduces the number of trigonometric functions from eight to three and requires only seven integrators. Furthermore, the memory requirement is limited to 96 samples, which is lower than that of the MAF-PLL and I-αβ -EPLL methods. Note that the memory requirements are calculated by considering the unit delay ($z^{-1}$) and the delay adjusted according to the MAF’s window-length ($z^{-N}$) in the MAF structure, as shown in Fig 5 [36]. The measured execution time of the proposed E²PLL is approximately 14.85 μs, which is significantly lower than that of I-*αβ*-EPLL [41] (51.93 μs), even though both demonstrate the highest filtering capability among the evaluated methods. These results indicate that the proposed E²PLL achieves an effective balance between computational complexity, memory requirement, execution speed, and filtering capability. Consequently, the proposed algorithm is well suited for real-time implementation in DSP-based DSTATCOM applications, where fast dynamic response and strong disturbance rejection are simultaneously required.

**Table 4:** Comparative evaluation of the computational complexity and implementation requirements of PLLs.

PLL Method	Math operators	Trigonometric Function	Number of Integrator	Memory usage (samples)	Execution Time (μs)	Filtering capability
EPLL [33]	53	8	12	–	4.81	Moderate
DSOGI-PLL [38]	31	4	6	–	2.1	Moderate
MAF-PLL [35]	24	4	2	102	13.07	High
TPSF-EPLL [40]	28	4	3	–	1.33	Poor
I-αβ -EPLL [41]	37	4	5	102	51.93	Very High
Proposed E^2^PLL	54	3	7	96	14.85	Very High

### Computation of PCC voltage magnitude and unit voltage templates

In the DSTATCOM control algorithm shown in Fig 2, the PCC voltage magnitude and unit voltage templates (UVTs) are required to determine the reference source currents ($i_{sa}^{*}$, $i_{sb}^{*}$, and$i_{sc}^{*}$). Additionally, in-phase UVTs and their quadrature templates are generated to synchronize the DSTATCOM output voltages with the SEIG terminals. For this purpose, the line-to-line voltages are measured, and the amplitude of PCC voltages is computed as:


$$\begin{array}{c} V_{t}=\sqrt{\frac{\text{2}}{\text{3}}(v_{sab}^{\text{2}}+v_{sbc}^{\text{2}}+v_{sca}^{\text{2}})} \end{array}$$ (11)

It should be noted that (11) is derived under the assumption of voltage balance conditions. However, under unbalanced conditions, the DSTATCOM actively compensates for voltage imbalances at the PCC. Therefore, there is no issue with using the amplitude value calculated from (11) for reference current generation.

Using (11), the in-phase UVTs are calculated as:


$$\begin{array}{c} u_{pab}=\frac{v_{sab}}{V_{t}};\text{\textbackslash{}hspace\{0.33em}\}u_{pbc}=\frac{v_{sbc}}{V_{t}};\text{\textbackslash{}hspace\{0.33em}\}u_{pca}=\frac{v_{sca}}{V_{t}} \end{array}$$ (12)

The quadrature UVTs are then obtained as:


$$\begin{array}{c} \begin{array}{c} u_{qab}=\frac{-u_{pbc}+u_{pca}}{\sqrt{\text{3}}}\\ u_{qbc}=\frac{3u_{pab}+u_{pbc}-u_{pca}}{2\sqrt{\text{3}}}\\ u_{qca}=\frac{-3u_{qab}+u_{qbc}-u_{qca}}{2\sqrt{\text{3}}} \end{array} \end{array}$$ (13)

As illustrated in Fig 2, the amplitude of load currents ($I_{Lm}$), in-phase and quadrature UVTs, zero crossing detectors (ZCDs), and sample-and-hold (S&H) circuits are used to determine the fundamental active components ($I_{Lap}$, $I_{Lbp}$, and $I_{Lcp}$) and the fundamental reactive components ($I_{Laq}$, $I_{Lbq}$, and $I_{Lcq}$) of the load currents.

### Fundamental active current components estimation

It is essential to determine the active current components to maintain the frequency of SEIG voltages at the reference value (50 Hz). Therefore, this frequency estimated by the proposed E^2^PLL is compared with its reference value, and the frequency error is processed through a PI controller. The output of the PI controller ($I_{fp}$) is the active current component to be compensated. The fundamental average active current component ($I_{Lpavg}$) is calculated as in (14). The magnitude of the active component of the reference current ($I_{spt}$) is then obtained by subtracting the$I_{fp}$ from the $I_{Lpavg}$ as in (15).


$$\begin{array}{c} I_{Lpavg}=\frac{I_{Lap}+I_{Lbp}+I_{Lcp}}{\text{3}} \end{array}$$ (14)

$$\begin{array}{c} I_{spt}=I_{Lpavg}-I_{fp} \end{array}$$ (15)

As shown in the reference current calculation (RCC) unit in Fig 2, the reference active current component for each phase ($i_{sap}^{*}$, $i_{sbp}^{*}$, and $i_{scp}^{*}$) is obtained by multiplying the $I_{spt}$ by in-phase UVTs as follows:


$$\begin{array}{c} i_{sap}^{*}=I_{spt}u_{pa};\text{\textbackslash{}hspace\{0.33em}\}i_{sbp}^{*}=I_{spt}u_{pb};\text{\textbackslash{}hspace\{0.33em}\}i_{scp}^{*}=I_{spt}u_{pc} \end{array}$$ (16)

### Fundamental reactive current components estimation

To keep the peak value of SEIG voltages at the desired level, it is required to obtain the reactive current components. For this purpose, the difference between the amplitude of the PCC voltage ($V_{t}$) and its reference value ($V_{tref}$) is applied as input to the PI controller. The output of this controller provides the reactive current component to be compensated ($I_{vtq}$). The fundamental average reactive current component ($I_{Lqavg}$) is computed as in (17). The magnitude of the reactive component of the reference current ($I_{sqt}$) is then calculated by subtracting the $I_{Lqavg}$ from the$I_{vtq}$ as in (18).


$$\begin{array}{c} I_{Lqavg}=\frac{I_{Laq}+I_{Lbq}+I_{Lcq}}{\text{3}} \end{array}$$ (17)

$$\begin{array}{c} I_{sqt}=I_{vtq}-I_{Lqavg} \end{array}$$ (18)

As a result, as illustrated in Fig 2, the reference reactive current component for each phase ($i_{saq}^{*}$, $i_{sbq}^{*}$, and $i_{scq}^{*}$) is determined by multiplying the$I_{sqt}$ by quadrature UVTs as:


$$\begin{array}{c} i_{saq}^{*}=I_{sqt}u_{qa};\text{\textbackslash{}hspace\{0.33em}\}i_{sbq}^{*}=I_{sqt}u_{qb};\text{\textbackslash{}hspace\{0.33em}\}i_{scq}^{*}=I_{sqt}u_{qc} \end{array}$$ (19)

### Reference current estimation

Based on (16) and (19), the reference source currents ($i_{sa}^{*}$, $i_{sb}^{*}$, and$i_{sc}^{*}$) at the output of the RCC unit, as shown in Fig 2, are obtained as:


$$\begin{array}{c} i_{sa}^{*}=i_{sap}^{*}+i_{saq}^{*};\text{\textbackslash{}hspace\{0.33em}\}i_{sb}^{*}=i_{sbp}^{*}+i_{sbq}^{*};\text{\textbackslash{}hspace\{0.33em}\}i_{sc}^{*}=i_{scp}^{*}+i_{scq}^{*} \end{array}$$ (20)

The reference source currents in (20) are compared with the measured SEIG currents in switching signal generation (SSG) unit, and the current error is processed using a carrier-based PWM technique. At the output of SSG, the switching signals (S_1_, S_2_, …, S_6_) are generated as shown in Fig 2. The generated switching signals are applied to the IGBTs on VSC in Fig 1.

## Experimental results and discussion

**Fig 14:**
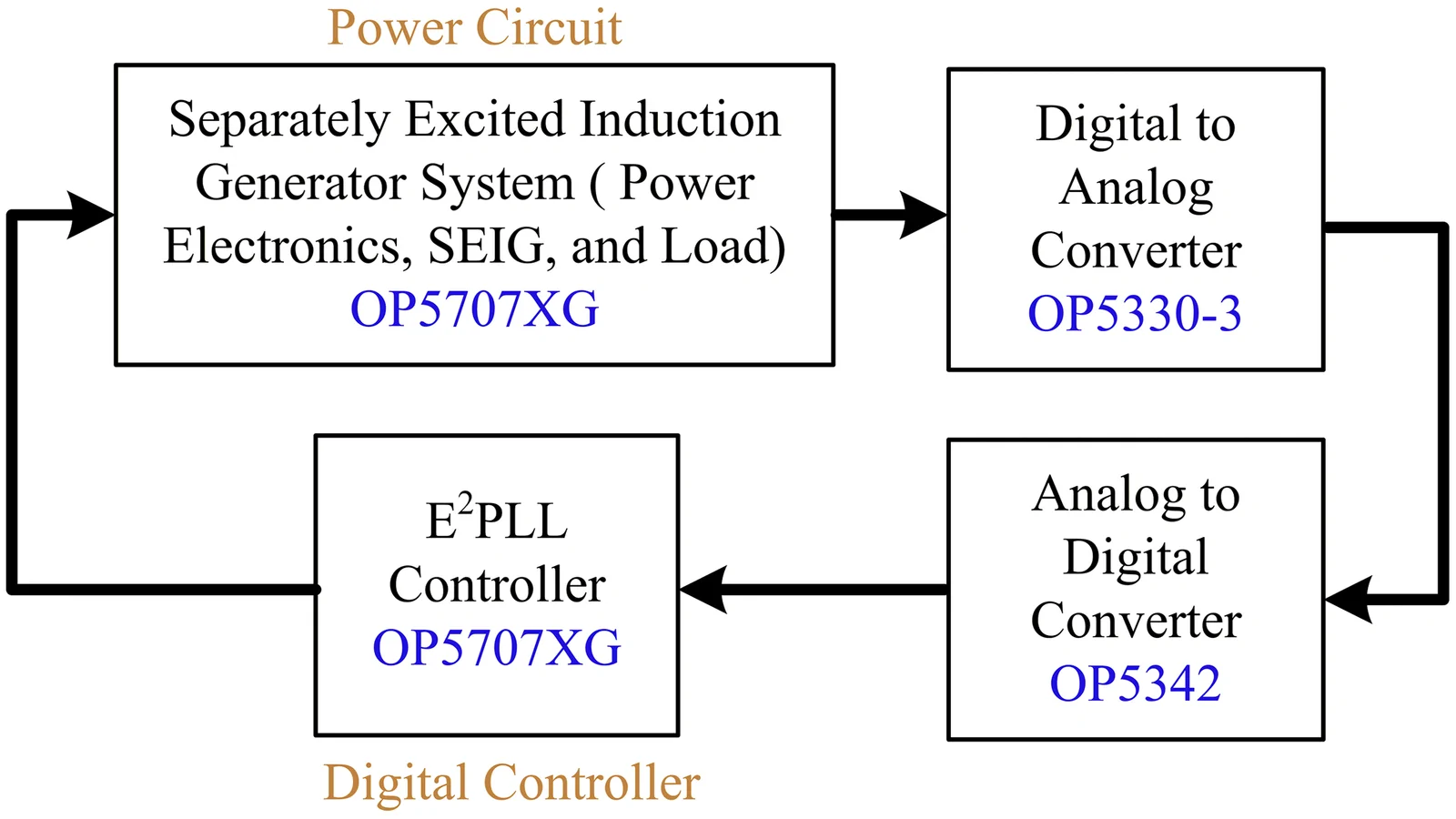
Real-time experimental setup based on OPAL-RT system.

This section evaluates the performance of the proposed E^2^PLL-based control method for DSTATCOM through an OPAL-RT system. The OPAL-RT is a digital simulator proven to accurately represent physical systems and capable of performing real-time hardware-in-the-loop (HiL) tests [42–44]. Using the OPAL-RT, complex power systems, microgrids, motor drives, and renewable energy applications can be run in real-time. Besides, the developed control algorithms can be safely tested on the OPAL-RT platform before being tested on actual hardware. Fig 14 shows the experimental setup based on the OPAL-RT system. In this study, the power stage (SEIG, STATCOM, etc.) and the proposed control algorithm are performed in real-time on the OP5707XG simulator, which has a sampling frequency of 20 kHz. The load current signals, DSTATCOM current signals, SEIG voltage and current signals are transformed into analog signals via the OP5330−3 digital-to-analog converter (DAC) module. The analog signals are subsequently sampled via the OP5342 analog-to-digital converter (ADC) module for utilization within the proposed controller. The experimental results are displayed on a digital storage oscilloscope.

The proposed E^2^PLL-based control method for DSTATCOM is tested under various challenging operating conditions, including nonlinear load, unbalanced and nonlinear load, and single-phase open-circuit fault scenarios. In addition, dynamic load variations are introduced during the tests to evaluate the transient performance of the system. These test scenarios comprehensively represent practical operating conditions such as harmonic distortions, load imbalances, fault conditions, and dynamic disturbances commonly encountered in real-world applications. Details of the tests conducted to demonstrate the effectiveness of the proposed method are provided below.

**Test-1:** To create a nonlinear load condition, a three-phase diode-rectified DC load is connected to the SEIG’s common connection point (PCC). During the test, the load is increased, so that the system is tested not only under load conditions containing harmonic distortion but also under increasing load demand.

**Test-2:** In this test, an unbalanced and nonlinear load bank consisting of 10 Ω, 25 Ω, and 15 Ω resistors is connected in parallel with the nonlinear load in the first test. This provides both unbalanced and nonlinear load conditions. Furthermore, the load is increased, and the test is continued.

**Test-3:** In this test, the proposed control method is evaluated in the case of a single-phase open-circuit fault. A resistive load of 8 Ω is connected to phases a and c, while no load is connected to phase b.

**Fig 15:**
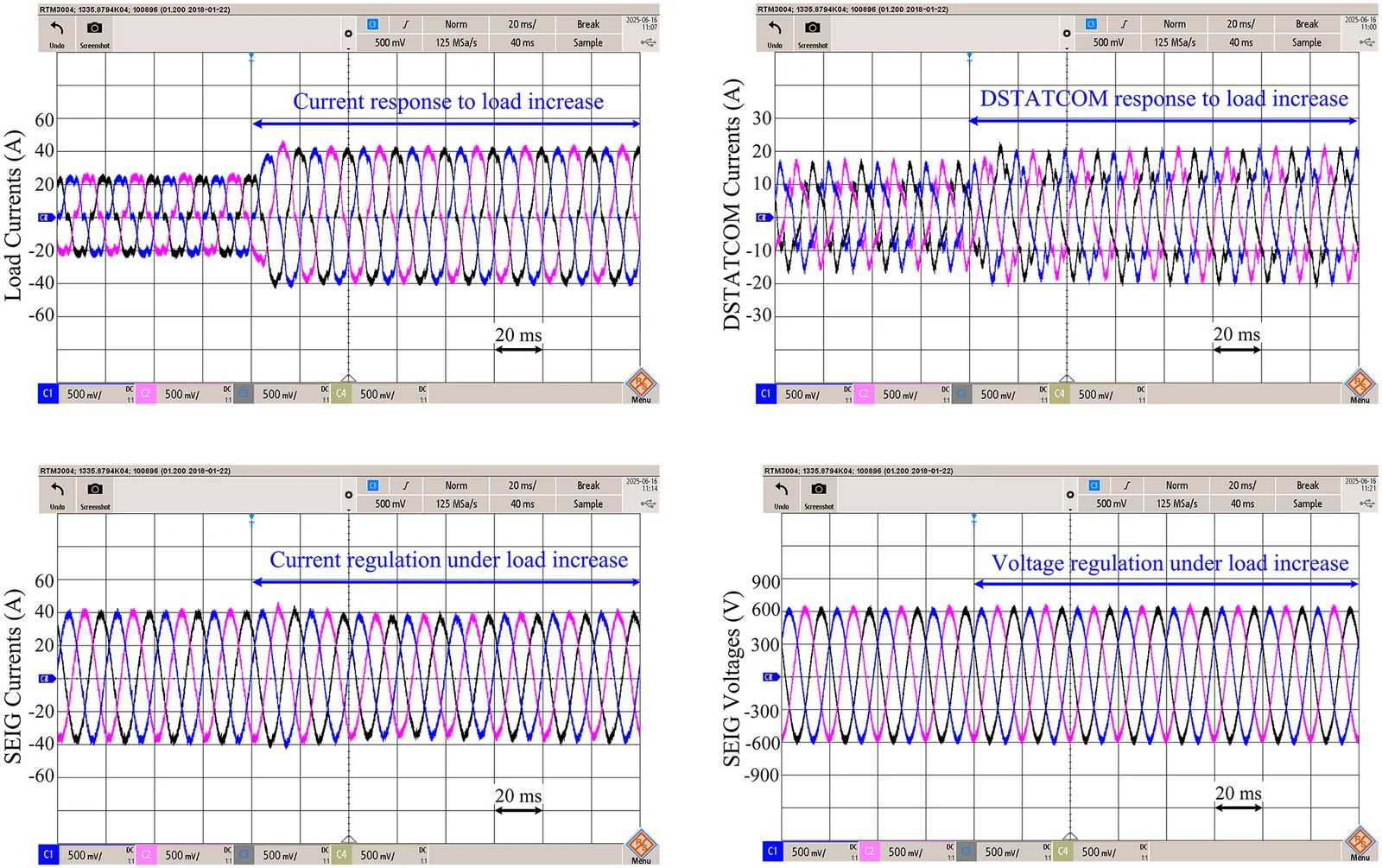
Performance of proposed E^2^PLL-based DSTATCOM control method under nonlinear load (a) Load currents; (b) DSTATCOM currents; (c) SEIG currents; (d) SEIG voltages, where (a) is top-left, (b) is top-right, (c) is bottom-left, and (d) is bottom-right.

**Fig 16:**
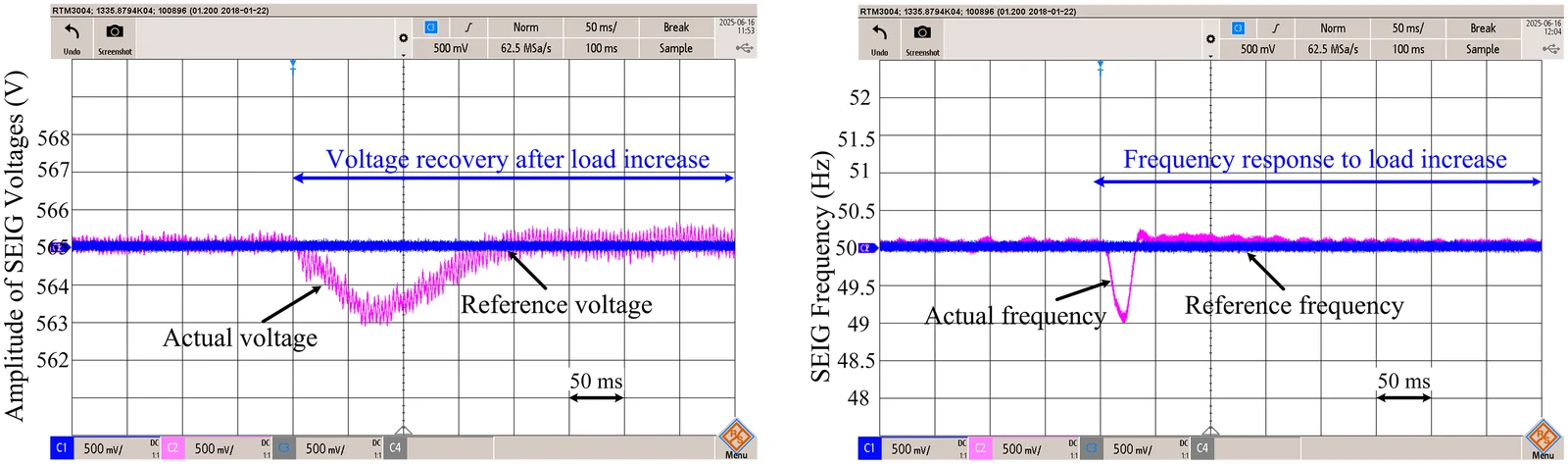
Performance of proposed E^2^PLL-based DSTATCOM control method under nonlinear load (a) Amplitude of SEIG voltages; (b) SEIG frequency, where (a) is left and (b) is right.

Figs 15, 18, and 21 demonstrate the load currents, DSTATCOM currents, SEIG currents, and SEIG voltages of the proposed E^2^PLL-based control method for three test conditions, respectively. Figs 16, 19, and 22 show the amplitude and frequency of the SEIG voltages for the same test conditions, respectively.

In Test-1, as shown in Fig 15a, harmonic components occur in the load currents due to nonlinear loads. As seen in this figure, as the load is increased during the test, the currents drawn by the load are approximately doubled. Fig 15b shows the compensation currents produced by the DSTATCOM to suppress harmonics in the system. The load increase has also increased the currents injected into the system by the DSTATCOM, as it increases the amount of active and reactive power demanded. These DSTATCOM currents are correctly generated thanks to the amplitude and frequency information of the load currents obtained by the proposed E^2^PLL-based control algorithm. Figs 15c and 15d show the currents and voltages of the SEIG, respectively. As can be seen from the figures, despite the significant increase in the nonlinear load, the current and voltage values remain balanced and sinusoidal. The results obtained clearly demonstrate that, thanks to the E^2^PLL algorithm, the DSTATCOM effectively eliminates harmonic distortion by generating accurate compensation currents and adapts to the load increase.

Fig 16 shows the peak voltage and frequency of the DSTATCOM-based SEIG system operating under nonlinear load. As seen in Fig 16a, despite the sudden increase in the load, thanks to the proposed control method, there is only a temporary 2 V drop in the amplitude of the SEIG voltages, and after a few periods, it accurately follows the reference value (565 V). Fig 16b shows the frequency response of the SEIG voltages. In the face of a challenging situation such as a load increase, there is a short-term drop of up to 1 Hz in frequency, but this deviation is also quickly corrected thanks to the control algorithm, and the SEIG frequency returned to the reference value (50 Hz). In conclusion, Test-1 demonstrates that the E^2^PLL-based DSTATCOM control algorithm regulates the SEIG’s voltage and frequency with high accuracy and stability, despite load increases under nonlinear load conditions.

**Fig 17:**
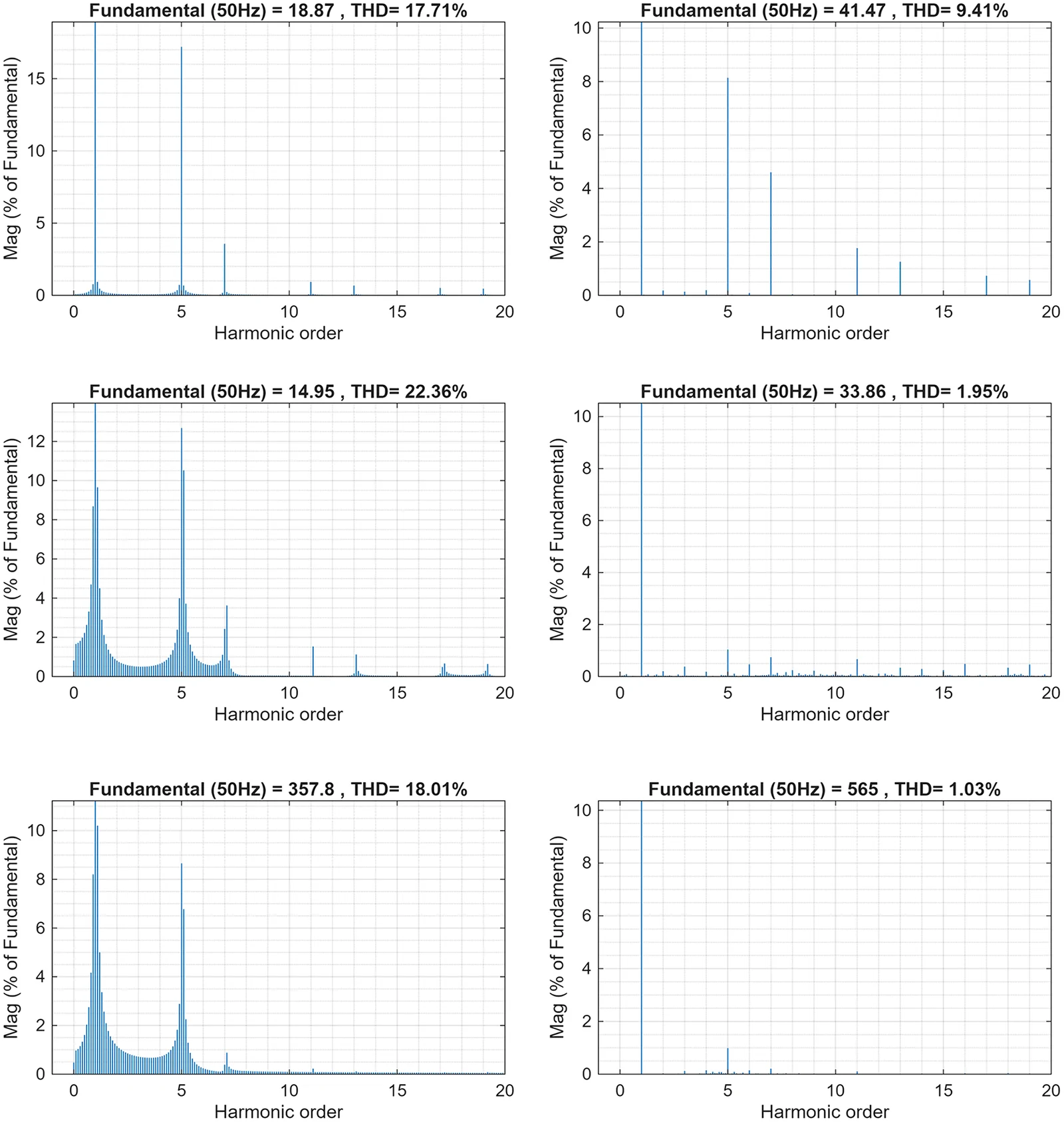
Harmonic spectrum before and after compensation by the proposed E^2^PLL-based DSTATCOM controller under nonlinear load. (a) Harmonic spectrum of load current before compensation; (b) Harmonic spectrum of load current after compensation; (c) Harmonic spectrum of SEIG current before compensation; (d) Harmonic spectrum of SEIG current after compensation; (e) Harmonic spectrum of SEIG voltage before compensation; (f) Harmonic spectrum of SEIG voltage after compensation, where (a) is top-left, (b) is top-right, (c) is middle-left, (d) is middle-right, (e) is bottom-left, and (f) is bottom-right.

The harmonic spectrums under nonlinear load condition are presented in Fig 17, while the corresponding quantitative performance indices are summarized in Table 5. Before compensation, the nonlinear load produces significant low-order current harmonics, resulting in SEIG current and voltage THD values of 22.36% and 18.01%, respectively. After compensation, the dominant harmonic components are strongly suppressed, reducing these values to 1.95% and 1.03%. In parallel, the power factor increases from 0.88 to 0.99, and the terminal voltage regulation error decreases from 36.67% to 0.17%, as shown in Fig 16a.

**Table 5:** Power quality and voltage regulation performance of the proposed E²PLL-based DSTATCOM controller under different operating conditions.

Test Conditions	Compensation Status	Load current THD (%)	SEIG current THD (%)	SEIG voltage THD (%)	Power factor	Voltage regulation error (%)
**Test-1:Nonlinear load**	Without compensation	17.71	22.36	18.01	0.88	36.67
	With compensation	9.41	1.95	1.03	0.99	0.17
**Test-2: Unbalanced and nonlinear load**	Without compensation	11.99	11.99	11.17	0.96	12.87
	With compensation	5.35	1.47	0.53	0.99	0.12
**Test-3: Single-phase open-circuit fault**	Without compensation	11.78	11.78	11.78	0.76	7.26
	With compensation	0.50	1.65	0.65	0.99	0.14

**Fig 18:**
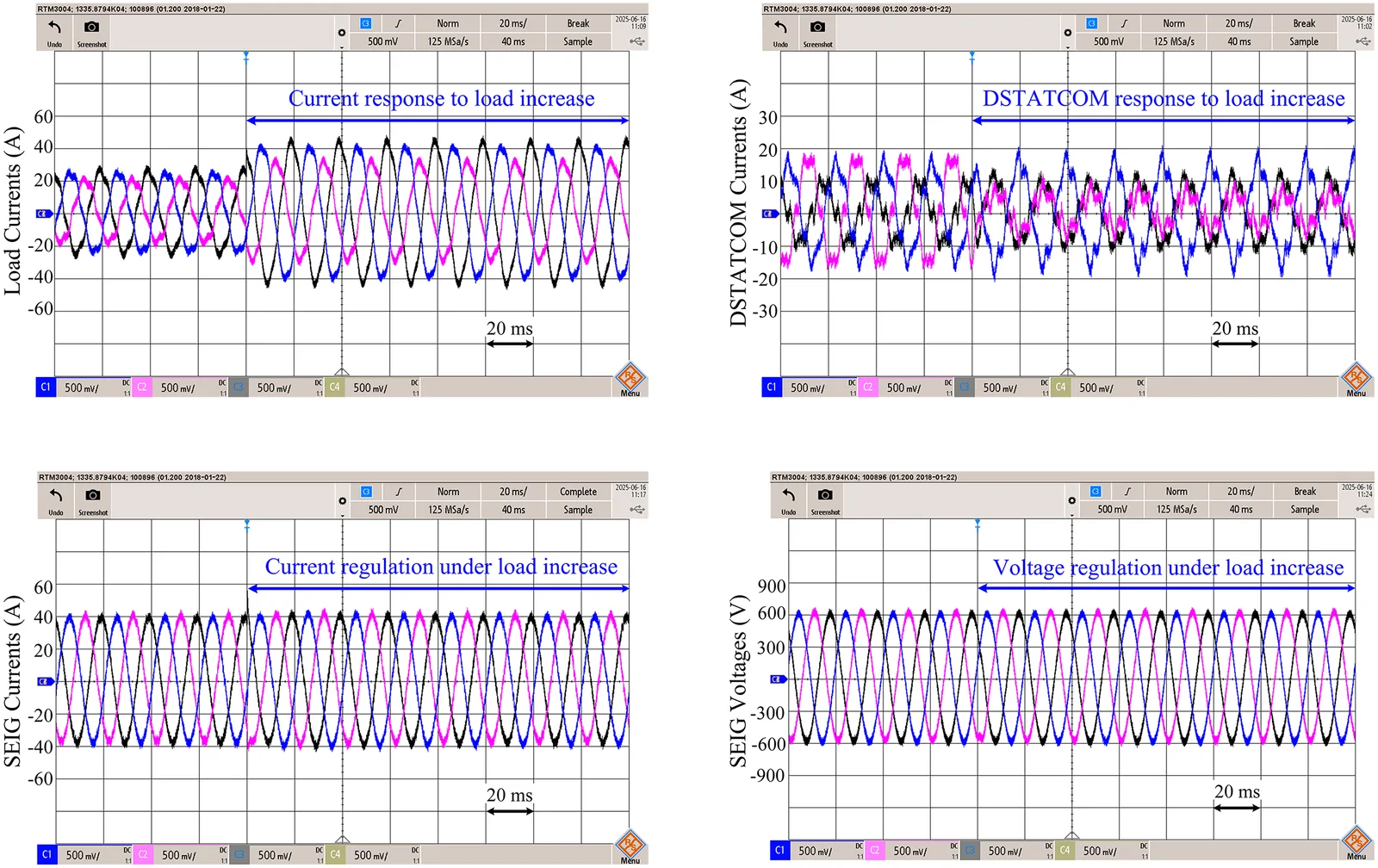
Performance of proposed E^2^PLL-based DSTATCOM control method under unbalanced and nonlinear load (a) Load currents; (b) DSTATCOM currents; (c) SEIG currents; (d) SEIG voltages, where (a) is top-left, (b) is top-right, (c) is bottom-left, and (d) is bottom-right.

Fig 18a shows the unbalanced and nonlinear load currents in Test-2. Due to the nature of the load, both harmonics and imbalances occur in these currents. In addition to the complex load profile, the load level is also increased during the test. This significantly complicates the voltage and frequency regulation of the SEIG. Fig 18b shows the currents injected into the system by the DSTATCOM. As observed in Figs 18c and 18d, thanks to the proposed E^2^PLL-based control method, the SEIG currents and voltages remain balanced and maintain their sinusoidal form even with the load increase. Consequently, Test-2 confirms that the proposed control method demonstrates effective performance even under extreme load conditions.

**Fig 19:**
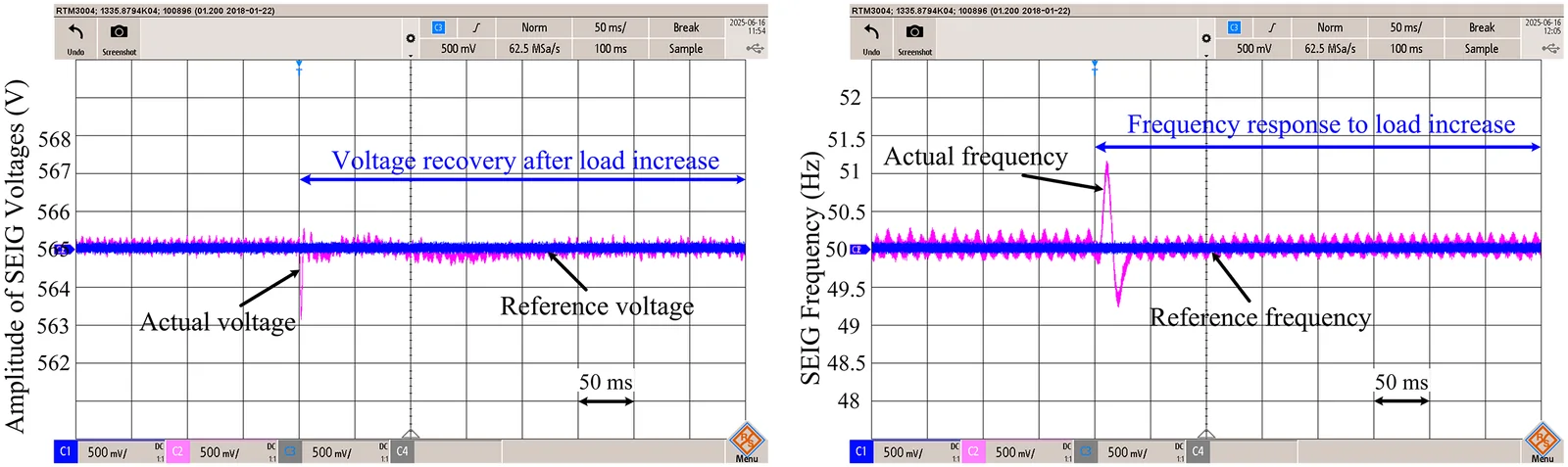
Performance of proposed E^2^PLL-based DSTATCOM control method under unbalanced and nonlinear load (a) Amplitude of SEIG voltages; (b) SEIG frequency, where (a) is left and (b) is right.

Fig 19 shows the variations in peak voltage and frequency of the DSTATCOM-based SEIG system under unbalanced and nonlinear loads. As seen in Fig 19a, during load changes, the amplitude of the SEIG voltages momentarily drops below the reference value for a very short period. However, the sudden decrease in voltage level is quickly compensated for thanks to the fast-dynamic performance of the E^2^PLL control algorithm. Fig 19b shows the change in the frequency of the SEIG voltages in response to changing conditions. As observed, although there is an overshoot of approximately 1.25 Hz in frequency during the transient state, the frequency settles to the reference value after a few periods. This test proves that the E^2^PLL algorithm successfully controls the DSTATCOM even under complex load conditions involving simultaneous imbalance and harmonic distortion, maintaining both the SEIG voltage and frequency stably at their reference values.

**Fig 20:**
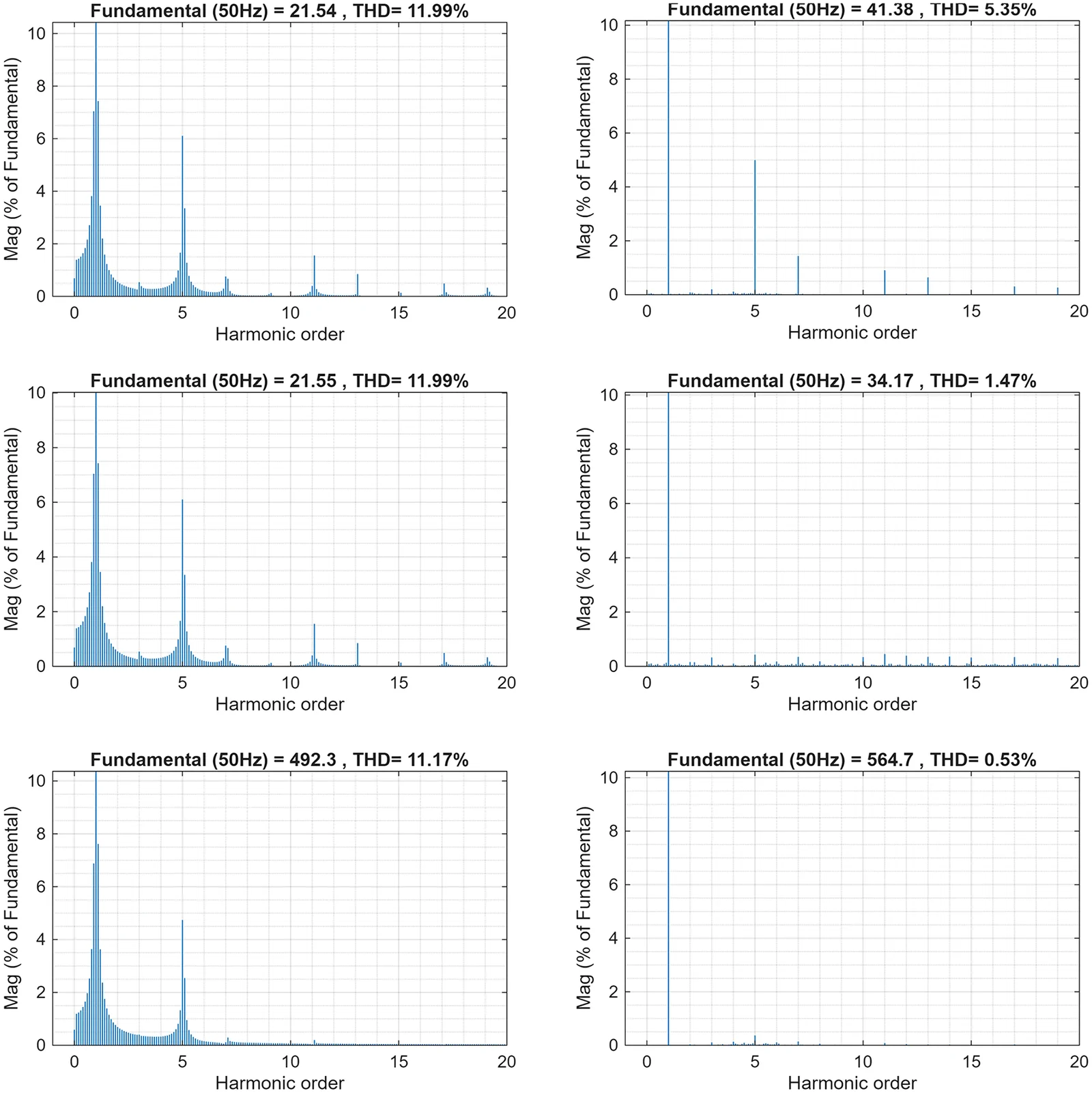
Harmonic spectrum before and after compensation by the proposed E^2^PLL-based DSTATCOM controller under unbalanced and nonlinear load. (a) Harmonic spectrum of load current before compensation; (b) Harmonic spectrum of load current after compensation; (c) Harmonic spectrum of SEIG current before compensation; (d) Harmonic spectrum of SEIG current after compensation; (e) Harmonic spectrum of SEIG voltage before compensation; (f) Harmonic spectrum of SEIG voltage after compensation, **where (a) is top-left, (b) is top-right, (c) is middle-left, (d) is middle-right, (e) is bottom-left, and (f) is bottom-right**.

The harmonic spectrums corresponding to unbalanced and nonlinear load conditions are presented in Fig 20, whereas the associated quantitative results are provided in Table 5. As shown, the combined unbalanced and nonlinear loading introduces both harmonic distortion and phase imbalance. Before compensation, the SEIG current and voltage THD values are 11.99% and 11.17%, respectively. After compensation, these values are reduced to 1.47% and 0.53%. Furthermore, the power factor improves from 0.96 to 0.99, while the terminal voltage regulation error decreases from 12.87% to 0.12% (see Fig 19a), demonstrating effective compensation under simultaneous unbalanced and nonlinear load conditions.

**Fig 21:**
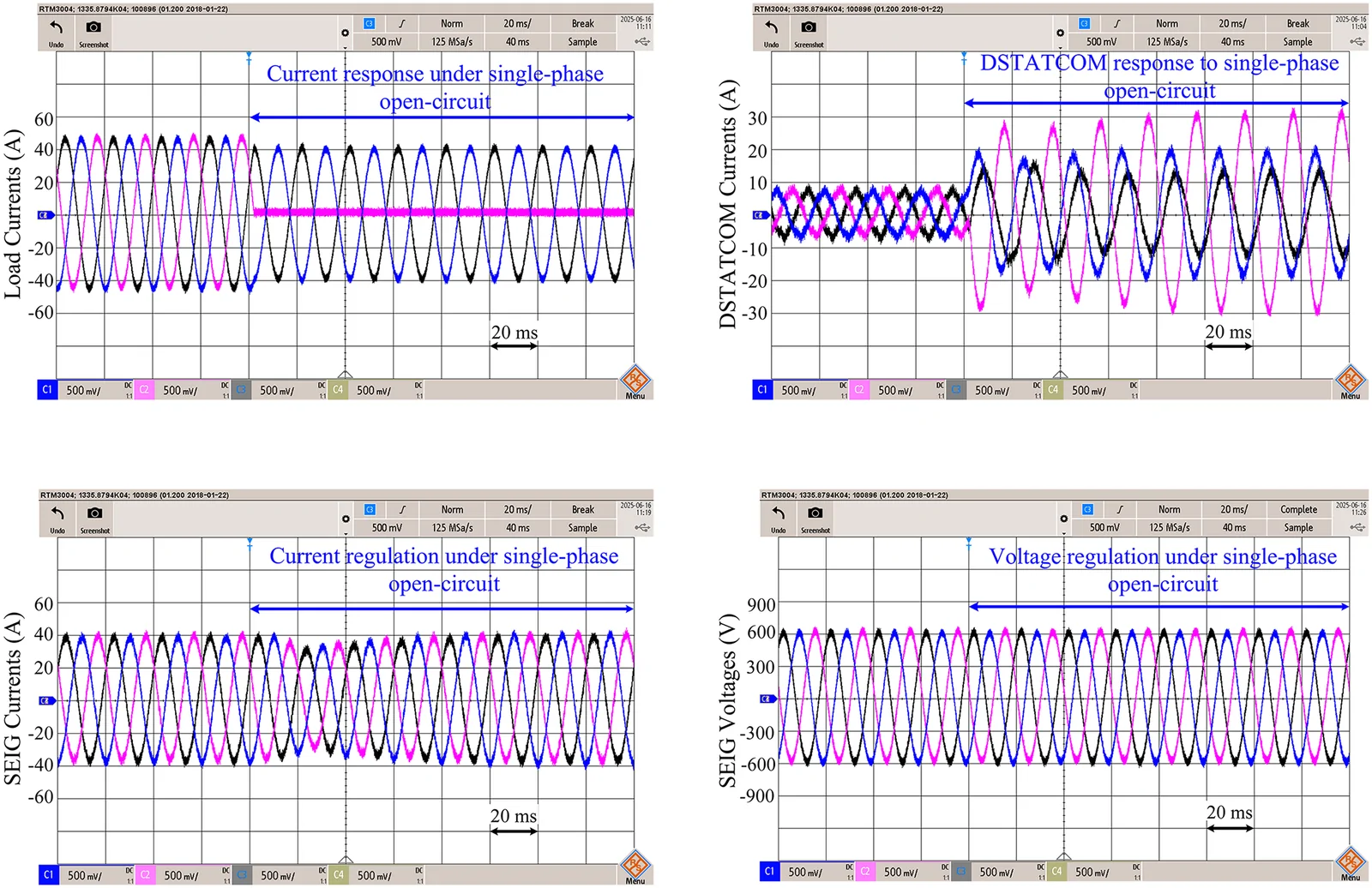
Performance of proposed E^2^PLL-based DSTATCOM control method under single-phase open-circuit fault (a) Load currents; (b) DSTATCOM currents; (c) SEIG currents; (d) SEIG voltages, where (a) is top-left, (b) is top-right, (c) is bottom-left, and (d) is bottom-right.

In Test-3, the performance of the proposed method is evaluated under single-phase open-circuit condition. As clearly shown in Fig 21a, no load is connected to phase b during the test, thereby reducing the current drawn from this phase to zero. Consequently, the power drawn by the load naturally decreases, causing the peak value of the current drawn from phases a and c to drop by approximately 5 A. Fig 21b shows the change in the compensation currents generated by the DSTATCOM during load variation. As seen, the DSTATCOM generates current signals with an asymmetric waveform in response to the severe imbalance condition that occurs. Figs 21c and 21d show the currents and voltages of the SEIG, respectively. As can be seen from these figures, despite the open-circuit in phase b, the SEIG currents and voltages maintain their three-phase balanced and sinusoidal form in steady-state thanks to the proposed control method. This situation demonstrates that the SEIG continues its efficient power production even under serious imbalance conditions such as a single-phase open circuit fault. Furthermore, this test demonstrates the effectiveness of the proposed E^2^PLL-based control method in DSTATCOM control.

**Fig 22:**
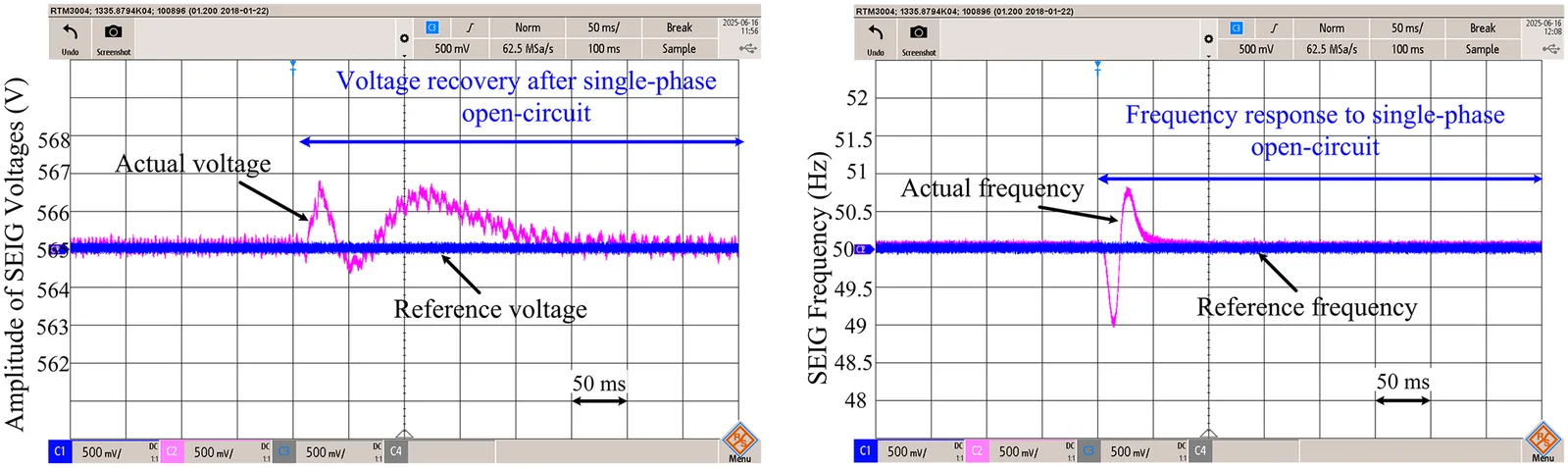
Performance of proposed E^2^PLL-based DSTATCOM control method under single-phase open-circuit fault (a) Amplitude of SEIG voltages; (b) SEIG frequency, where (a) is left and (b) is right.

Fig 22 shows the variations in the peak value and frequency of the SEIG voltages in a single-phase open-circuit condition. As seen in Fig 22a, an overshoot of approximately 1.8 V in amplitude occurs in the transient state. Thanks to the superior performance of the proposed E^2^PLL method, it follows its reference value after a few periods. Fig 22b shows that there is a 1 Hz drop in the frequency of the SEIG voltages in the fault condition, but shortly afterwards, the frequency accurately follows its reference value. The results obtained, particularly in Test-3, highlight the success of the E^2^PLL-based control algorithm. Even under severe imbalance conditions, the proposed E^2^PLL-based DSTATCOM algorithm ensures that SEIG currents and voltages are obtained in a symmetrical and balanced manner. Furthermore, transient deviations in the peak value and frequency of the SEIG voltages are quickly and successfully eliminated by the superior characteristics of the E^2^PLL structure.

**Fig 23:**
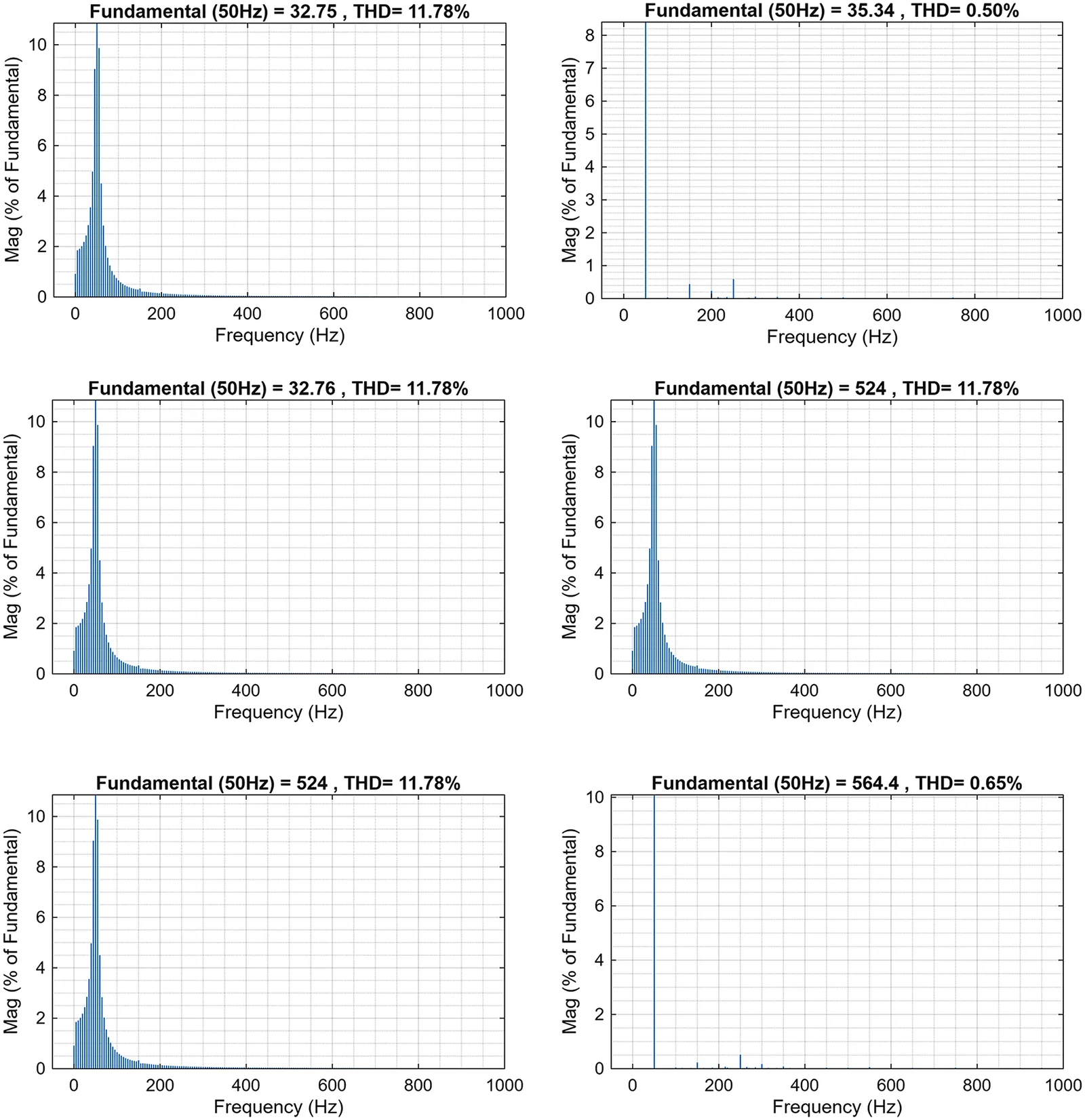
Harmonic spectrum before and after compensation by the proposed E^2^PLL-based DSTATCOM controller under single-phase open-circuit fault. (a) Harmonic spectrum of load current before compensation; (b) Harmonic spectrum of load current after compensation; (c) Harmonic spectrum of SEIG current before compensation; (d) Harmonic spectrum of SEIG current after compensation; (e) Harmonic spectrum of SEIG voltage before compensation; (f) Harmonic spectrum of SEIG voltage after compensation, **where (a) is top-left, (b) is top-right, (c) is middle-left, (d) is middle-right, (e) is bottom-left, and (f) is bottom-right**.

The harmonic spectrums under the single-phase open-circuit fault are demonstrated in Fig 23, and the corresponding quantitative performance metrics are given in Table 5. Before compensation, the fault condition causes severe current imbalance and harmonic distortion, resulting in SEIG current and voltage THD values of 11.78%. After compensation, these values decrease to 1.65% and 0.65%. Moreover, the power factor improves from 0.76 to 0.99, while the terminal voltage regulation error is reduced from 7.26% to 0.14% (see Fig 22a), confirming the effectiveness of the proposed controller even under severe fault conditions.

### Robustness evaluation of proposed control method under practical operating disturbances

To further evaluate the robustness of the proposed E²PLL-based DSTATCOM controller under practical operating conditions, additional studies are carried out considering measurement noise, DC-link voltage fluctuations, and wind-speed variations under nonlinear load condition. These disturbances represent common challenges encountered in stand-alone wind energy conversion systems.

**Fig 24:**
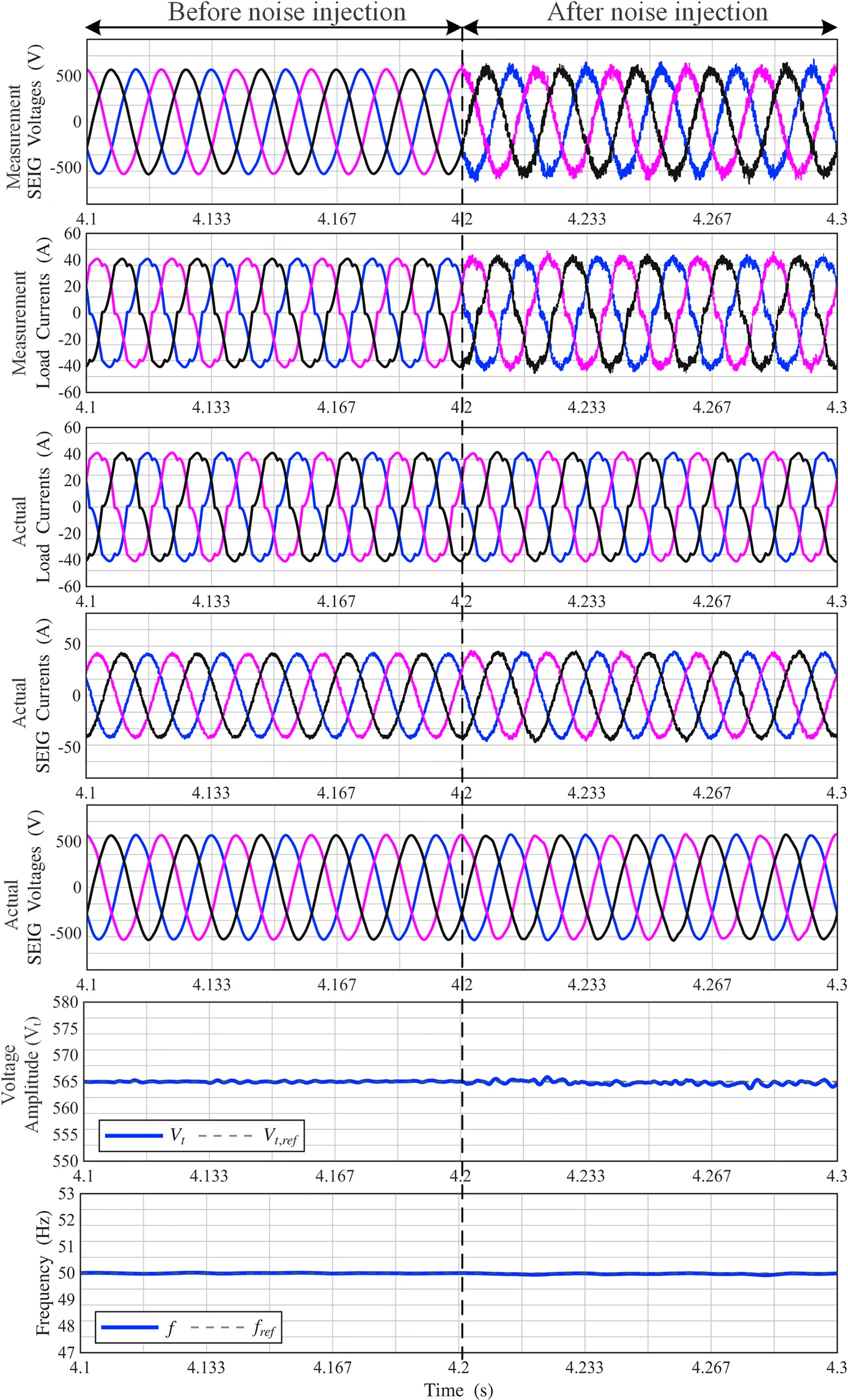
Robustness evaluation of the proposed E²PLL-based DSTATCOM before and after measurement noise injection under nonlinear load condition.

Firstly, in order to investigate the robustness of the proposed E²PLL-based DSTATCOM controller against measurement noise, band-limited white Gaussian noise is intentionally injected into the measured three-phase voltages and load currents at t = 4.2 s. The corresponding results are presented in Fig 24. As expected, the injected noise significantly distorts the measured voltage and current waveforms after the disturbance is applied. However, the proposed controller continues to accurately extract the fundamental components, maintaining balanced SEIG currents and nearly sinusoidal SEIG terminal voltages throughout the test. Despite the severe degradation in the measured signals, the terminal voltage amplitude remains tightly regulated around its reference value of 565 V, while the estimated system frequency stays very close to the nominal value of 50 Hz with only negligible variations. Furthermore, the noisy measured load current THD reaches 10.56%, whereas the THD values of the SEIG terminal voltage and SEIG current remain limited to only 2.32% and 2.38%, respectively. These results indicate that the proposed E²PLL-based controller effectively suppresses the influence of measurement noise and preserves accurate synchronization, voltage regulation, and frequency regulation under noisy sensing conditions.

**Fig 25:**
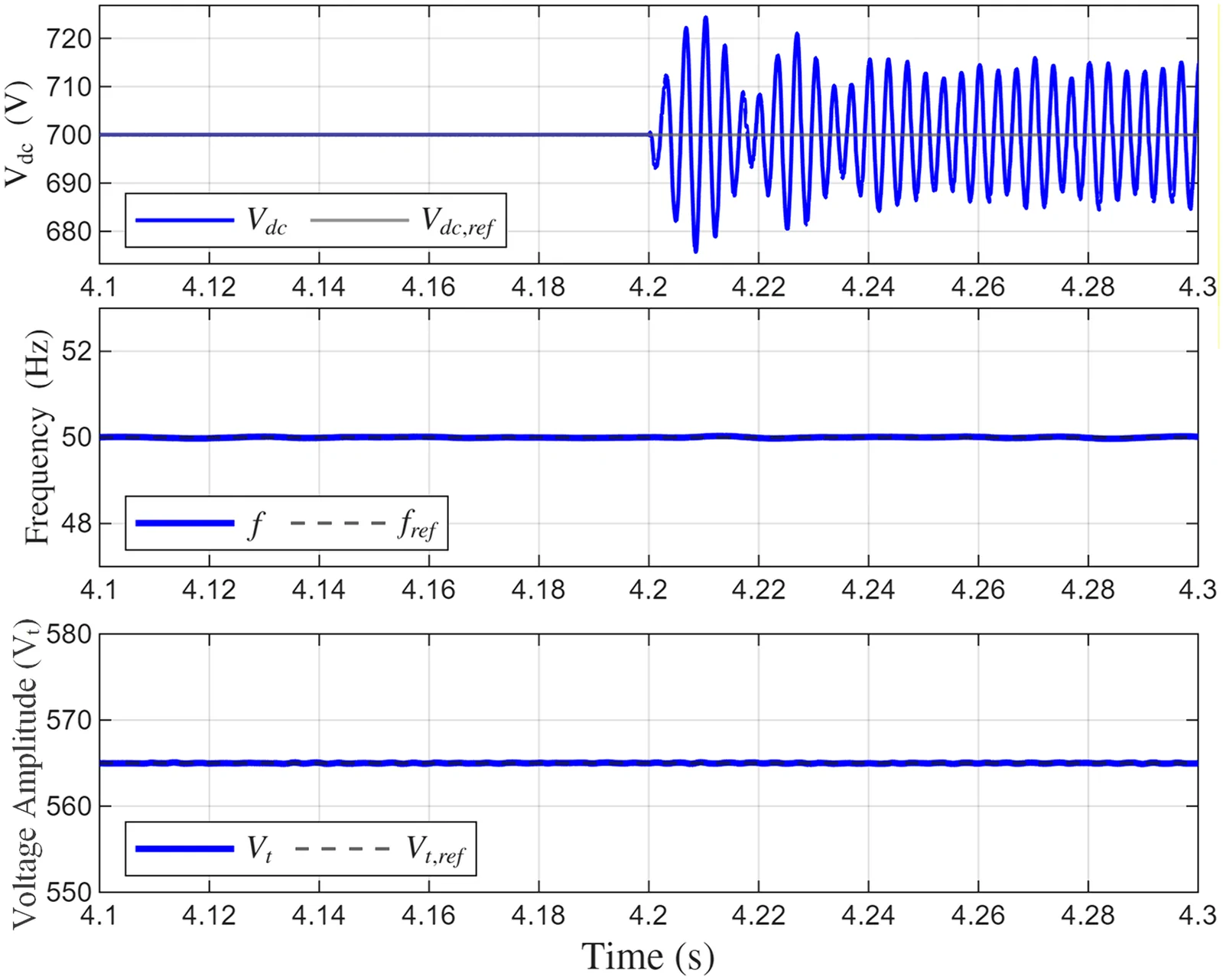
Robustness evaluation of the proposed E²PLL-based DSTATCOM controller under DC-link voltage fluctuations under nonlinear load condition.

To examine the robustness of the proposed E²PLL-based DSTATCOM controller against DC-link voltage fluctuations, an intentional disturbance is introduced into the DC-link voltage at t = 4.2 s. As illustrated in Fig 25, the applied disturbance produces significant oscillations around the nominal DC-link voltage of 700 V. Despite these fluctuations, the proposed controller successfully maintains the system frequency at its nominal value of 50 Hz and preserves the SEIG terminal voltage close to its reference value of 565 V. The results show that even under pronounced DC-link voltage ripple, the proposed controller maintains stable operation and accurate voltage-frequency regulation, confirming its robustness to DC-link voltage fluctuations.

**Fig 26:**
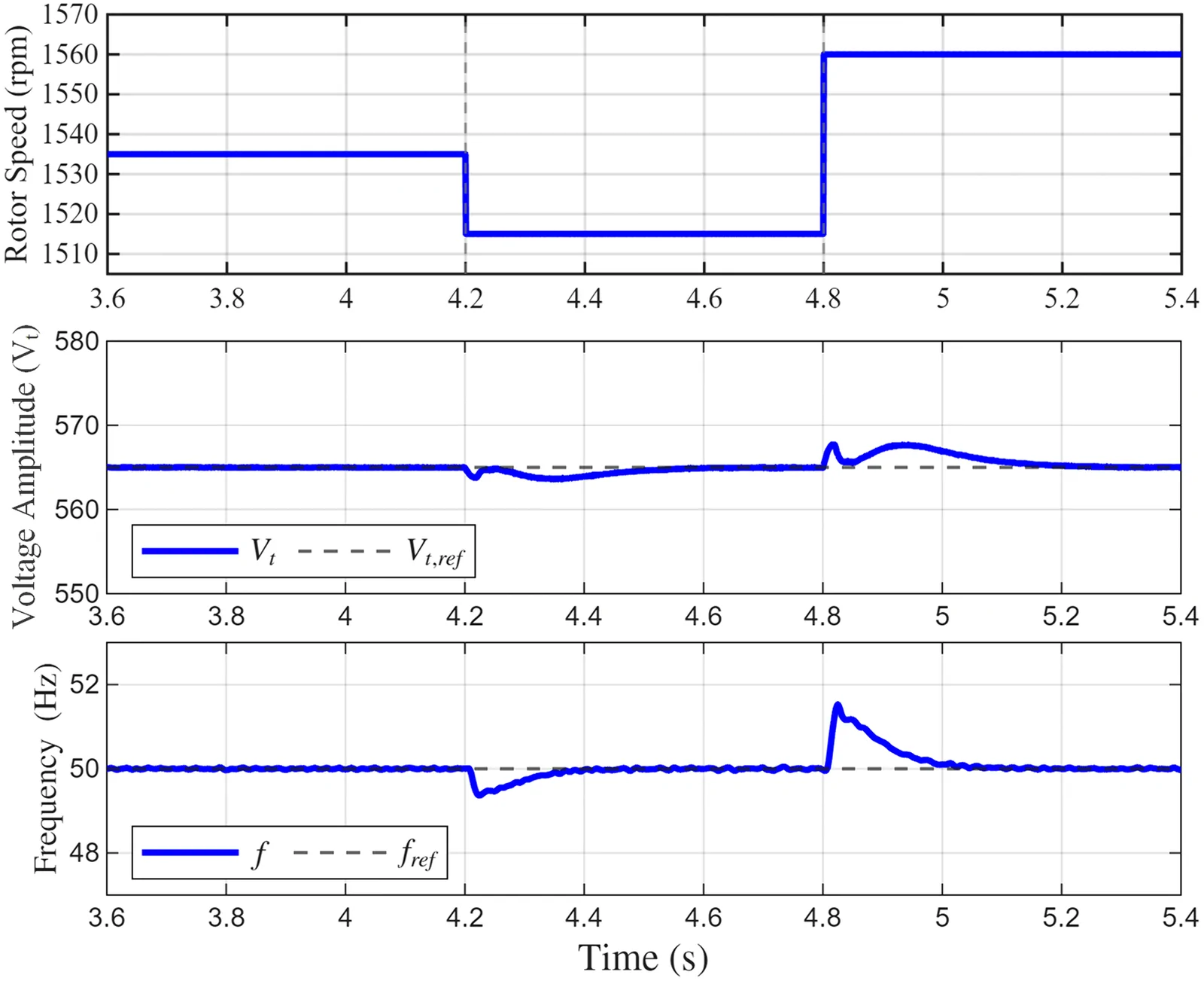
Dynamic responses of the proposed E²PLL-based DSTATCOM controller under sudden wind-speed variations under nonlinear load condition.

Lastly, to evaluate the robustness of the proposed E²PLL-based DSTATCOM controller against wind-speed variations, the rotor speed is intentionally varied to emulate realistic changes in the available wind power. As depicted in Fig 26, the rotor speed is initially maintained at 1535 rpm and then reduced to 1515 rpm at t = 4.2 s, representing a sudden decrease in wind speed. Subsequently, at t = 4.8 s, the rotor speed is increased to 1560 rpm to emulate a sudden increase in wind speed. The reduction in rotor speed causes only a slight transient decrease in both the terminal voltage and system frequency. Nevertheless, the proposed controller rapidly compensates for the disturbance, restoring both variables close to their reference values without sustained oscillations. Likewise, the subsequent increase in rotor speed results in a limited overshoot in the voltage and frequency responses, which is effectively damped within a short settling period. Despite the larger positive speed variation, the proposed controller maintains stable operation and quickly re-establishes the nominal operating conditions. Unlike a single operating-point evaluation, the proposed controller is validated under both sudden decreases and increases in rotor speed, thereby demonstrating robust performance under realistic wind-speed variations. Overall, the results confirm that the proposed E²PLL-based DSTATCOM controller effectively preserves terminal-voltage and frequency regulation under varying mechanical input conditions, highlighting its suitability for standalone SEIG-based wind energy conversion systems.

## Comparative analysis of proposed approach with existing methods

Table 6 presents the numerical results of comparative analysis of the proposed E^2^PLL-based control method with four existing DSTATCOM control approaches, namely MAF-based method [45], SOGI-based method [46], EPLL-based method [15], and FH-PLL-based method [47]. THD values of the load current, SEIG current, and SEIG voltage are evaluated under three operating scenarios such as nonlinear load, unbalanced and nonlinear load, and single-phase open-circuit fault. It should be noted that although all control methods are tested under identical load conditions, THD values of the load currents seem slightly different in Table 6. The observed differences arise due to the interaction between the control strategies and the SEIG voltage regulation, which indirectly affects the harmonic content of the load currents.

**Table 6:** Numerical results of comparative analysis.

Test Conditions→	Nonlinear load			Unbalanced and nonlinear load			Single-phase open-circuit fault		
THD Parameters→	Load current THD	SEIG current THD	SEIG voltage THD	Load current THD	SEIG current THD	SEIG voltage THD	Load current THD	SEIG current THD	SEIG voltage THD
**Proposed E** ^**2**^ **PLL-based method**	9.41%	1.95%	1.03%	5.35%	1.47%	0.53%	0.50%	1.65%	0.65%
**MAF-based method [45]**	9.56%	3.27%	2.29%	5.75%	2.20%	1.11%	0.75%	2.65%	1.01%
**SOGI-based method [46]**	9.63%	3.45%	1.96%	5.85%	3.51%	2.09%	0.93%	4.53%	1.53%
**EPLL-based method [15]**	9.56%	3.24%	1.60%	5.82%	2.70%	1.22%	0.75%	4.20%	1.02%
**FH-PLL-based method [47]**	9.47%	2.56%	1.51%	5.81%	2.42%	1.74%	0.83%	2.70%	1.06%

Under nonlinear load condition, THD value of the SEIG current for the proposed E^2^PLL-based method is 1.95%, which is lower than the values obtained using MAF-based method (3.27%), SOGI-based method (3.45%), EPLL-based method (3.24%), and FH-PLL-based method (2.56%). Similarly, THD of the SEIG voltage is reduced to 1.03% with the proposed method, while these values are 2.29%, 1.96%, 1.60%, and 1.51%, respectively, for the MAF-based, SOGI-based, EPLL-based, and FH-PLL-based methods. THD values of all methods are within the limits of IEEE 519–2022 standard (<5%) [48]. As can be understood, the proposed method provides improved harmonic suppression capability under nonlinear load condition.

Under unbalanced and nonlinear load condition, the proposed method achieves a SEIG current THD of 1.47%, which is significantly lower than those of MAF-based method (2.20%), SOGI-based method (3.51%), EPLL-based method (2.70%), and FH-PLL-based method (2.42%). Moreover, in terms of SEIG voltage THD, the proposed method provides the best performance with a value of 0.53%, outperforming all existing methods (1.11%, 2.09%, 1.22%, and 1.74%). Consequently, the proposed E^2^PLL-based control method exhibits strong imbalance and harmonic rejection capability.

In single-phase open-circuit condition, the proposed method continues to demonstrate robust performance. The SEIG current THD is limited to 1.65%, which is considerably lower than those of MAF-based method (2.65%), SOGI-based method (4.53%), EPLL-based method (4.20%), and FH-PLL-based method (2.70%). Similarly, the SEIG voltage THD is maintained at 0.65%, compared to 1.01%, 1.53%, 1.02%, and 1.06% for the existing methods.

Overall, the results demonstrate that THD values achieved using the proposed E^2^PLL-based method are consistently lower than those of the existing methods and meet the limits specified by the IEEE 519–2022 standard [48]. These findings confirm that the proposed method provides superior harmonic and imbalances mitigation capability and robust performance under various challenging operating conditions.

**Fig 27:**
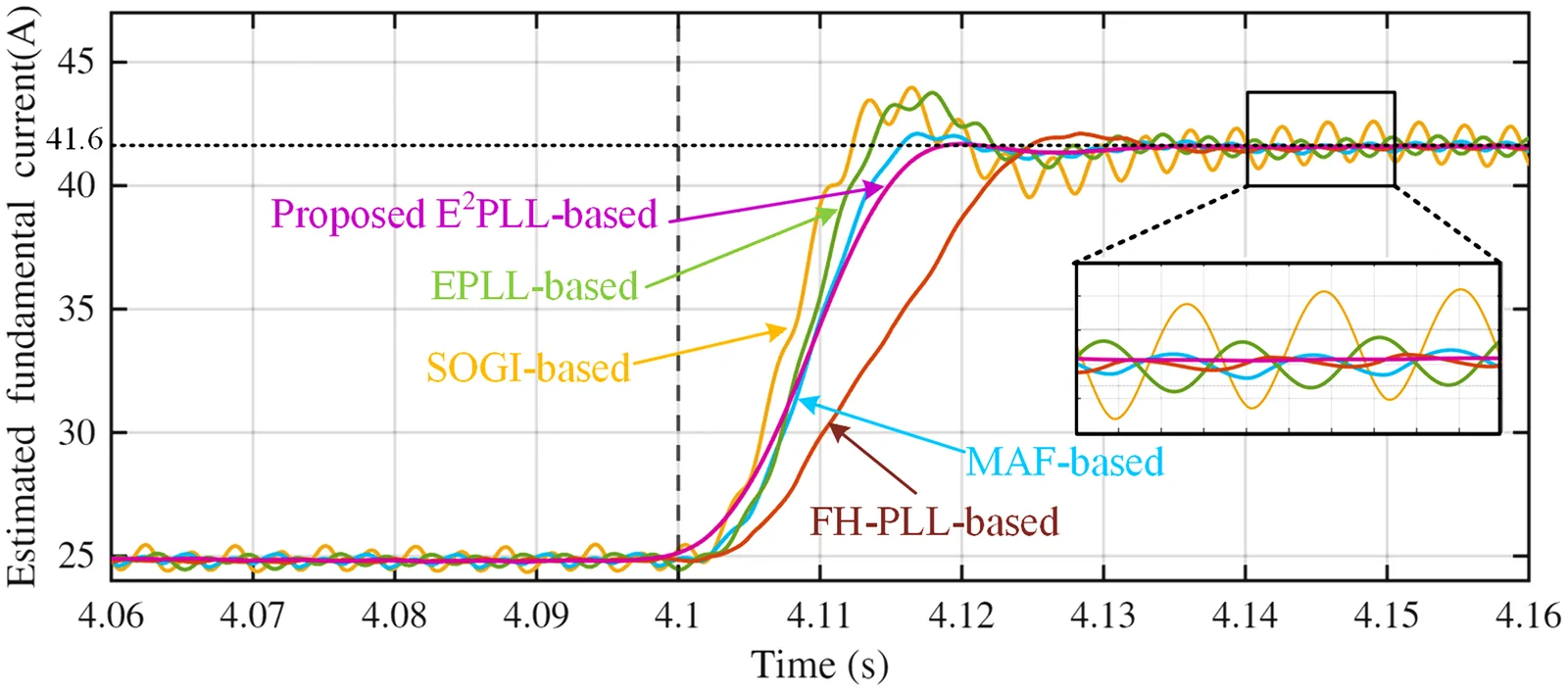
Dynamic comparison of the estimated fundamental current responses of different DSTATCOM control methods under a sudden load increase.

In addition to the THD analysis, the performances of the DSTATCOM algorithms are analyzed under both transient and steady-state conditions in order to provide a comprehensive quantitative evaluation. Fig 27 shows the estimated fundamental current responses of the methods under a sudden load change. As observed, the proposed E²PLL-based method exhibits the fastest transient response with the shortest settling time, the lowest overshoot, and the smallest steady-state oscillation among all compared methods. These observations are quantitatively summarized in Table 7, where the proposed control method achieves a settling time of 16.95 ms, an overshoot of 0.209%, a steady-state error of 0.0067 A, and the lowest current RMS error of 0.0079 A.

**Table 7:** Comprehensive performance comparison of DSTATCOM control methods under transient load conditions.

Estimated Signals→	Fundamental Current Component				Terminal Voltage Component (*V*_*t*_)				
Performance Metrics→	Settling time (ms)	Overshoot (%)	Steady-state error (A)	RMS error (A)	Minimum terminal voltage (V)	Voltage dip (%)	Settling time (ms)	Steady-state error (V)	RMS error (V)
**Proposed E** ^**2**^ **PLL-based method**	16.95	0.209	0.0067	0.0079	561.98	0.535	0	0.071	0.088
**MAF-based method [45]**	28.05	1.201	0.0937	0.1071	551.75	2.345	111.90	0.131	0.159
**SOGI-based method [46]**	39.85	5.699	0.4930	0.5541	549.78	2.694	138.05	0.273	0.355
**EPLL-based method [15]**	39.15	5.185	0.2243	0.2538	548.23	2.969	151.30	0.448	0.548
**FH-PLL-based method [47]**	29.40	1.220	0.1510	0.1623	556.84	1.444	74.15	0.154	0.185

**Fig 28:**
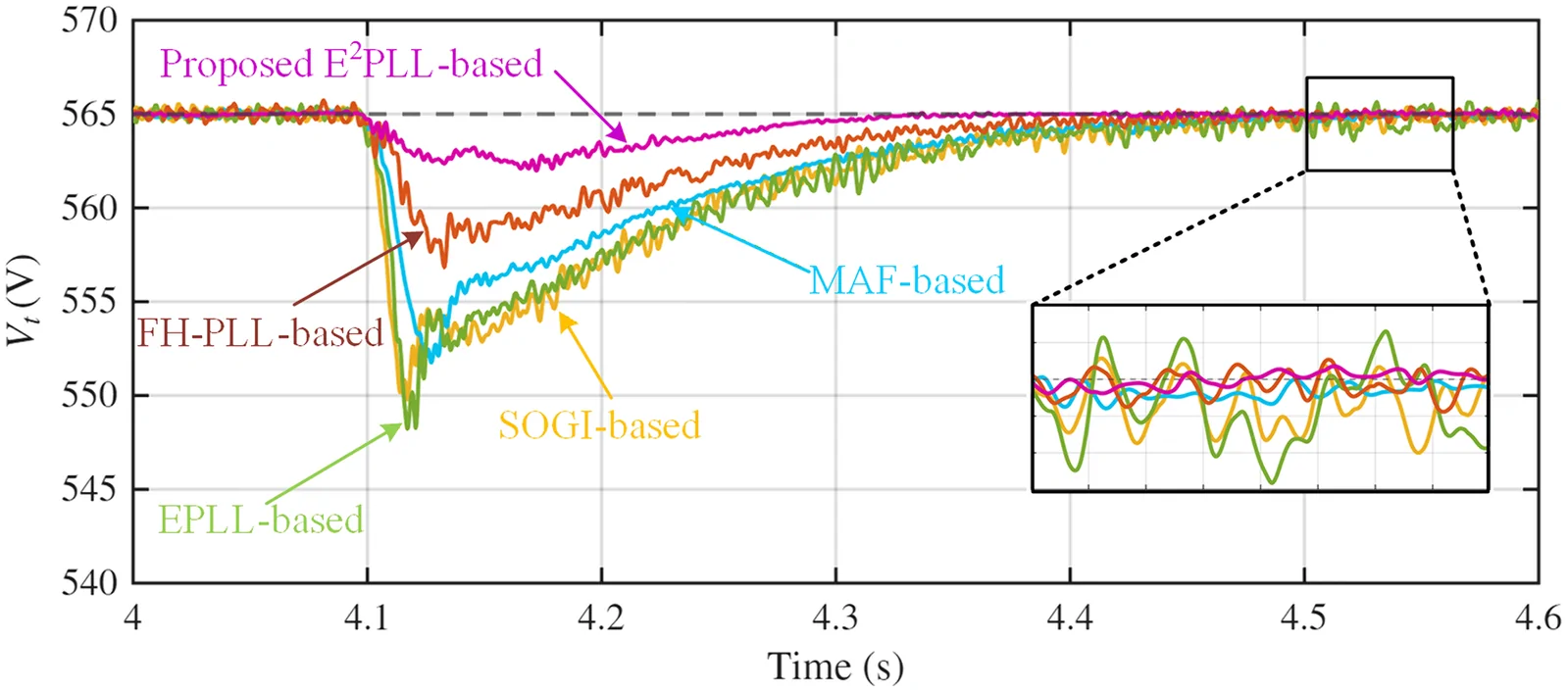
Dynamic comparison of the terminal voltage responses of different DSTATCOM control methods under a sudden load increase.

Additionally, Fig 28 illustrates the corresponding terminal voltage responses of the DSTATCOM control method employing different PLL algorithms during the same transient event. Compared with the other control methods, the proposed E²PLL-based method significantly reduces the terminal-voltage dip and maintains the voltage within the ± 1% tolerance band throughout the transient period. As summarized in Table 7, the proposed method limits the voltage dip to 0.535%, while achieving the lowest voltage steady-state error (0.071 V) and voltage RMS error (0.088 V).

The comparison presented in Tables 6 and 7 demonstrates the superior performance of the proposed E²PLL-based DSTATCOM controller under the considered test conditions. Furthermore, recent DSTATCOM control strategies – such as DSOGI-PLL-based methods [49], SOGI-FLL-based approaches [50], adaptive filtering methods [24–29], disturbance observer (DO)-based controllers [21], and artificial intelligence (AI)-based solutions [10] – are compared. Since these strategies differ not only in synchronization performance but also in harmonic rejection capability, dynamic response, computational burden, adaptability, and implementation complexity, a more comprehensive qualitative comparison is beneficial. Therefore, Table 8 summarizes the main characteristics, advantages, and limitations of representative DSTATCOM control strategies reported in the recent literature, providing a clearer positioning of the proposed controller with respect to existing approaches.

**Table 8:** Comparison of the proposed E²PLL-based DSTATCOM controller with recent DSTATCOM control strategies.

Control Strategy	Synchronization Accuracy	Harmonic Rejection	Dynamic Response	Computational Burden	Frequency Adaptability	Main Limitation
SRF-PLL-based [7]	Moderate	Moderate	Moderate	Low	Poor	Sensitive to harmonics and frequency variations
DSOGI-PLL-based [49]	High	High	Moderate	Moderate	Moderate	DC-offset sensitivity
SOGI-FLL-based [50]	High	High	High	Moderate	High	Frequency estimation slower during abrupt transients
Adaptive Filter-based [24–29]	High	Very High	Moderate	Moderate	High	Performance depends on gain adaptation
Disturbance Observer (DO)-based [21]	High	High	High	High	High	Observer design complexity
ANN-based [10]	Very High	Very High	High	Very High	Excellent	Requires training data and high computational resources
Proposed E²PLL-based	Very High	Very High	Very High	Moderate	High	DC-offset sensitivity

As can be understood in Table 8, conventional PLL- and adaptive filter-based approaches generally provide satisfactory harmonic mitigation and synchronization performance; however, their dynamic response is often limited by filter tuning, convergence speed, or computational complexity. Disturbance observer- and ANN-based methods improve robustness under uncertain operating conditions but require more complex implementations and, in the case of ANN-based controllers, offline training and larger computational resources. In contrast, the proposed E²PLL-based controller achieves fast dynamic response, excellent harmonic rejection capability, and accurate synchronization while maintaining a relatively low computational burden and without requiring observer design or offline training.

## Conclusion

In this study, the performance of the proposed E^2^PLL-based DSTATCOM control algorithm for voltage and frequency regulation of islanded SEIG systems is evaluated. The effectiveness of the proposed algorithm is tested in detail using OPAL-RT-based experimental setup under nonlinear load, unbalanced and nonlinear load, and single-phase open-circuit fault conditions. The experimental results for three different test conditions are summarized as follows:

- In the first test, although the nonlinear load draws harmonic currents from the SEIG system, thanks to the proposed E^2^PLL-based control method, the compensation currents of the DSTATCOM ensure that the SEIG output currents and voltages are balanced and sinusoidal. Moreover, even if the load is increased during the test, the amplitude and frequency of the SEIG voltages follow the steady-state reference value with great accuracy.
- In the second test, although the unbalanced load connected in parallel to the nonlinear load causes both harmonic distortion and imbalance in the load currents, the voltage and frequency regulation of the SEIG is guaranteed thanks to the superior performance of the proposed control algorithm.
- In the third test, the single-phase open-circuit fault causes the load currents to become asymmetrical, but the proposed control method ensures that the SEIG output currents and voltages become balanced and symmetrical after a few periods. Additionally, the transient deviations in the amplitude and frequency of the SEIG voltages during the fault are quickly cancelled.

In addition to the comparative evaluation with MAF-based, SOGI-based, EPLL-based and FH-PLL-based control methods, comparisons with recent DSTATCOM control strategies have been presented, demonstrating that the proposed E²PLL-based controller offers an effective balance between harmonic rejection capability, dynamic response, computational complexity, and implementation simplicity. Furthermore, additional robustness studies under practical operating conditions, including wind-speed variations, measurement noise, and DC-link voltage ripple, confirm that the proposed controller maintains stable voltage regulation, accurate frequency estimation, and satisfactory power-quality performance. These results demonstrate the suitability of the proposed E²PLL-based DSTATCOM controller for real-time implementation in stand-alone SEIG-based wind energy systems.

In future studies, integration with renewable energy sources and implementation of maximum power point tracking (MPPT)-based control strategies will be addressed to further enhance the energy efficiency of the system.
